# Insights into the pathophysiology of DFNA10 hearing loss associated with novel *EYA4* variants

**DOI:** 10.1038/s41598-020-63256-5

**Published:** 2020-04-10

**Authors:** Matias Morín, Lucía Borreguero, Kevin T Booth, María Lachgar, Patrick Huygen, Manuela Villamar, Fernando Mayo, Luis Carlos Barrio, Luciana Santos Serrão de Castro, Carmelo Morales, Ignacio del Castillo, Beatriz Arellano, Dolores Tellería, Richard J. H. Smith, Hela Azaiez, M. A. Moreno Pelayo

**Affiliations:** 1 Servicio de Genética, Ramón y Cajal Institute of Health Research (IRYCIS) and Biomedical Network Research Centre on Rare Diseases (CIBERER), 28034 Madrid, Spain; 2 Molecular Otolaryngology and Renal Research Laboratories, Department of Otolaryngology, Head & Surgery, University of Iowa, Iowa City, Iowa 52242 USA; 3 Harvard Medical School, Department of Neurobiology, Boston, Massachusetts 02115 USA; 4 Department of Otorhinolaryngology, Radboud University Nijmegen Medical Centre, Nijmegen, Netherlands; 5 Departamento de Investigación, Ramón y Cajal Institute of Health Research (IRYCIS), Unidad de Neurología Experimental, 28034 Madrid, Spain; 6 Servicio de Otorrinolaringología, Hospital Universitario Marqués de Valdecilla, 39008 Santander, Spain; 7 Servicio de Otorrinolaringología, Hospital Universitario Puerta de Hierro, Majadahonda, 28922 Madrid Spain

**Keywords:** Genetics, Genomics

## Abstract

The mutational spectrum of many genes and their contribution to the global prevalence of hereditary hearing loss is still widely unknown. In this study, we have performed the mutational screening of *EYA4* gene by DHLPC and NGS in a large cohort of 531 unrelated Spanish probands and one Australian family with autosomal dominant non-syndromic hearing loss (ADNSHL). In total, 9 novel *EYA4* variants have been identified, 3 in the EYA4 variable region (c.160G > T; p.Glu54*, c.781del; p.Thr261Argfs*34 and c.1078C > A; p.Pro360Thr) and 6 in the EYA-HR domain (c.1107G > T; p.Glu369Asp, c.1122G > T; p.Trp374Cys, c.1281G > A; p.Glu427Glu, c.1282-1G > A, c.1601C > G; p.S534* and an heterozygous copy number loss encompassing exons 15 to 17). The contribution of *EYA4* mutations to ADNSHL in Spain is, therefore, very limited (~1.5%, 8/531). The pathophysiology of some of these novel variants has been explored. Transient expression of the c-myc-tagged EYA4 mutants in mammalian COS7 cells revealed absence of expression of the p.S534* mutant, consistent with a model of haploinsufficiency reported for all previously described *EYA4* truncating mutations. However, normal expression pattern and translocation to the nucleus were observed for the p.Glu369Asp mutant in presence of SIX1. Complementary *in silico* analysis suggested that c.1107G > T (p.Glu369Asp), c.1281G > A (p.Glu427Glu) and c.1282-1G > A variants alter normal splicing. Minigene assays in NIH3T3 cells further confirmed that all 3 variants caused exon skipping resulting in frameshifts that lead to premature stop codons. Our study reports the first likely pathogenic synonymous variant linked to DFNA10 and provide further evidence for haploinsufficiency as the common underlying disease-causing mechanism for DFNA10-related hearing loss.

## Introduction

Sensorineural hearing loss (SNHL) is a common human genetic sensory defect. It is estimated that 1 of every 1000 children born in developed countries have hearing loss for which the genetic factors account for approximately two-thirds of the cases^1^. Among all the hereditary nonsyndromic SNHL, the autosomal recessive forms accounts for about 70–80% of cases and are typically congenital or prelingual. The population that suffers postlingual hearing loss, however, is significantly higher affecting 10% of the population by age 60, and 50% by age 80, although the proportion that is due to genetic causes has not been precisely determined^2^. The autosomal dominant hearing loss (ADNSHL) represents about 10–20% of all cases of hereditary SNHL; is characterized by high genetic and clinical heterogeneity and, in contrast to the recessive conditions, has a delayed onset (postlingual) and escapes the neonatal hearing screening. To date, more than 70 ADNSHL loci have been mapped and in 47 of them the responsible gene has been identified^3^. The contribution of many of these genes to the pool of ADNSHL is still unknown as comprehensive mutational screening has not yet been conducted in most populations worldwide. Amongst these genes is the transcriptional coactivator *EYA4* (Eyes Absent Homolog 4) belonging to the family of *Eya* genes, vertebrate orthologs (*Eya1–Eya4*) of the *Drosophila melanogaster* eyes absent gene (*Eya*). *EYA4* participates in the development of multiple organs, including the eye, pituitary gland, muscle, kidney, inner ear and heart^4^. Eya proteins contain an N-terminal variable region (VR) with transactivating function that includes the Neuronal helix-loop-helix transcription factor domain (Neuro-bHLH) and a highly conserved C-terminal Eya domain (eyaHR) that mediates interactions with members of the sine oculis family of proteins (Six1-Six6)^5^. Within each of these domains are several motifs that are essential for all EYA protein enzymatic activity^6^. More broadly, the EYA proteins are functionally multifaceted and belong to a highly conserved and complex network involving several families of transcriptional proteins that is known collectively as the Pax-Six-Eya-Dach network (PSEDN)^7,8^. The formation of the PSEDN is essential for proper development of many organs including the lungs, craniofacial skeleton, muscle, eyes, heart and ears^9^.

Since Eya proteins lack a DNA-binding domain they require to interact with the transcription factors Six and Dach to mediate the regulatory effects^10^. Six binding by Eya proteins is required for nuclear translocation^5^ where they participate in the regulation of gene expression^11,12^ through the phosphatase activity of the Eya domain that releases Dach-Six–mediated transcriptional repression of genes^13^.

In humans, mutations in two members of EYA gene family (*EYA1* and *EYA4*) cause syndromic and non-syndromic sensorineural hearing loss, respectively. Human *EYA1* (OMIM 601653) mutations cause branchiootorenal (BOR) syndrome (OMIM 113650), a disorder that presents with branchial arch defects including lachrymal duct abnormalities, preauricular fistulae, and hearing loss (mixed conductive and sensorineural), with abnormal shaped external ears, cochlear malformations, and renal anomalies^14^. *EYA4* mutations, in contrast, are responsible for the non-syndromic SNHL subtype DFNA10 (OMIM 601316), with no external ear or other craniofacial structures affected. So far, 38 variations in *EYA4* have been reported associated with DFNA10-related hearing loss. These include 11 frameshift variants^15–24^, 6 nonsense variants^15,20,25–28^, 16 missense variants^15–17,29–39^ and 5 splice-altering variant^15,40–43^. The age of onset of the hearing loss due to *EYA4* mutations shows a broad variation ranging from early childhood to adulthood^44^. Moreover, more complex mutations involving *EYA4* (large deletions affecting the gene structure) have been associated only with SNHL^15^ or with other clinical entities that encompass SNHL, dilated cardiomyopathy, and/or mental retardation^7,45–47^. Familiar reports of otofaciocervical syndrome (OTFS), without a cardiac phenotype have also been reported in patients carrying a large 3.7MB deletion in 6q23.1q23.2 that includes the entire *EYA4* gene^48^.

In this study, we have performed a comprehensive mutation screening in a large population of 531 ADSNHL Spanish patients and in an isolated Australian family. We have identified 9 novel genetic variations in *EYA4* gene associated with DFNA10 hearing loss: three missense, two nonsense, one frameshift, one splice-site, one silent mutation affecting splicing and one copy number loss encompassing exon 15 to exon 17. We have also investigated the physiopathological mechanism of four of these mutations by means of immunocytochemistry, western blotting and minigene assays.

## Patients and methods

### Sample collection and clinical evaluation

Patients and healthy relatives were recruited from the University Hospital Ramón y Cajal (Madrid-Spain) or the University of Iowa, Iowa City, Iowa, USA. This study was designed in compliance with the tenets of the Helsinki Declaration, and patient enrollment was approved by the ethics committee and the human research Institutional Review Boards of Hospital Ramón y Cajal (IRB number: 288-17) and University of Iowa (IRB number: 199701065). All participants provided written informed consent prior to their participation in this study.

A large cohort of 531 unrelated Spanish probands with ADNSHL was enrolled without any hearing loss phenotype preselection. Whole blood samples were obtained from all participants by venipuncture and genomic DNA was extracted by using standard procedures. Clinical history ruled out environmental factors as the cause of the hearing loss in the probands and physical examination did not reveal any evidence of syndromic features. No other clinically significant manifestations including balance or visual problems were reported by any of the affected individuals. Tympanometry indicated proper functioning of the middle ear. Pure tone audiometry with air and bone conduction at frequencies ranging 250-8.000 HZ was performed for all consenting families’ members according to standard protocols.

Family 10880 is a large four-generation Australian family segregating postlingual progressive ADNSHL. Clinical examination of the subjects was completed to exclude any additional and/or syndromic findings. Pure tone audiometry was performed to determine air conduction thresholds at 0.25, 0.5, 1, 2, 3, 4 and 8 kHz. Audiometric data were analyzed to determine the Age Related Typical Audiogram (ARTA) and Annual Threshold Deterioration (ATD) as previously described^49^. After obtaining written informed consent to participate in this study, saliva samples were obtained from 44 family members, 17 affected and 27 presumed unaffected, and genomic DNA was extracted following the manufacture’s protocol.

### Targeted genomic sequencing and data analysis

OtoNGSpanel V1 or OtoNGSpanel V2, custom gene panels were developed at the Hospital Ramón y Cajal. OtoNGSpanel V1 was based on HaloPlex technology to capture all exons and 25 bp of intronic flanking regions of 71 genes involved in hereditary hearing loss. The version 2 of the panel is based on IDT probes capture system and Sophia Genetics’ software and included 117 genes associated with SNHL. Version 1 and V2 were used to screen a cohort of 111 and 108 Spanish independent familial cases with ADSNHL, respectively. Sequencing of captured enriched-libraries was done on the Illumina MiSeq (Illumina, Inc., San Diego, CA). The sequence data were mapped against the human genome sequence (build GRCh37/hg19) and data analysis was performed using the Variant Studio and Sophia Genetics’ softwares, the latter enabling the copy number variation (CNV) analysis of the targeted exonic sequences. Variant prioritization was carried out using a custom filtering strategy.

OtoSCOPE® v6, a next-generation sequencing platform, was used to screen 109 genes implicated in NSHL and USH for possible mutations in two affected individuals, II.1 and II.13 of the Australian family 10880, as described^50–52^. Following sequencing on the Illumina HiSeq. 2000 (Illumina, Inc., San Diego, CA) using 100 bp paired-end reads, bioinformatics analysis was performed on a custom Galaxy pipeline running on the high-performance computing cluster at the University of Iowa as described^51,52^. Both samples underwent CNVs evaluation by assessing the read-depth ratio, as described^53^.

### DHPLC analysis and Sanger sequencing

In a Spanish initial cohort of 312 unrelated probands with ADSNHL, the exonic regions of *EYA4* gene [NM_004100.5] [MIM: 603550] were amplified using a standard protocol on a GeneAmp PCR® System 9700 (Applied Biosystems, Foster City, CA). PCR amplimers of each exon of the *EYA4* gene were screened for mutations by Denaturing High Performance Liquid Chromatography (DHPLC) on a WaveTM DNA fragment analysis system (TransgenomicTM, USA) according to manufacturer’s protocol. The different DHPLC heteroduplex profiles were characterized by sequencing a product of a second PCR amplification as previously described^54^.

Segregation analysis for the Spanish families and for the Australian family 10880 was achieved by PCR amplification of the respective target exons and flanking introns of *EYA4* followed by Sanger Sequencing. In all instances, sequencing was completed with a BigDyeTM v3.1 Terminator Cycle Sequencing kit (Applied Biosystems, Foster City, CA), according to the manufacturer’s instructions. The sequencing procedure was carried out in an ABI 3730 s sequencer (Perkin Elmer, Waltham, MA).

### Copy number variation confirmation by using Real Time PCR

*EYA4* DNA copy numbers in family S2532 was determined in an Applied Biosystems 7300 Real-time PCR system using SYBR premix Ex Taq (Takara) according to the manufacturer’s directions. All primers were designed with the Primer Express 2.0 software (Applied Biosystems). The following primers were used to amplify a fragment of *EYA4* containing the exon 16: 5′-CAACTGATGGCTTCCATGCA-3′ and 5′-TCTTACACCTGTTGGCAAACAAA-3′. As a chromosomal diploid reference we have amplified a region of *ALB* gene, which encodes the human albumin, by using the following primers: 5′-GCTGTCATCTCTTGTGGGCTGT-3′ and 5′-ACTCATGGGAGCTGCTGGTTC-3′. For each assay, we have included one replicate for each of the different DNA concentrations used (5 ng, 2 ng and 0.2 ng) in healthy relatives and patients. The cycling conditions were as follows: 2 min at 50 °C, 10 min at 95 °C, 40 cycles at 95 °C for 15 s, 59 °C for 30 s and 72 °C for 30 s. The threshold cycle (Ct) and the amplification efficiency (E) were obtained by using the 7300 system SDS software. Where E = 10^(−1/slope)^. Relative quantification (Q) was determined as follows: Q = E EYA4^(ΔCt EYA4)^/E ALB ^(ΔCt ALB)^, where ΔCt=mean Ct Healthy relatives-mean Ct patients. After performing relative quantification, statistical analysis was carried out by using SPSS software, version 15 (SPSS Inc.). To analyze the variance between the healthy relatives and patients, one-way ANOVA was used. A Pvalue of ≤0.001 was deemed significant.

### Expression of EYA4 mutants in COS7 cells

Expression vectors for *EYA4* (pE4, encoding the EYA4-HR domain) and SIX1 (pS1) wild type proteins were kindly provided by Prof. RJH Smith^55^. In order to generate a Wt bicistronic plasmid vector (pE4S1-Wt) to express both, the EYA4 and SIX1 proteins, a *Hind*III-*Xba*I fragment containing the c-myc encoded tag in frame with a distal DNA fragment that encodes the EYA4-HR domain (codons 322 to 616) was extracted from the pE4-HR vector and subsequently subcloned into the expression vector pS1. The final construct (pE4S1) was a bicistronic expression vector including the c-myc-tagget EYA4-HR domain driven by the CMV promoter and the HA-tagged SIX1 under the control of the EF-1 promoter (Suppl Fig. 1). The wild type individual (pE4-Wt) and bicistronic (pE4S1-Wt) vectors was then mutagenized using the QuickChange Site-Directed Mutagenesis Kit (Stratagene) to generate the mutated vectors (pE4_Glu369Asp_, pE4_Ser534*_, pE4S1_Glu369Asp_ and pE4S1_Ser534*_) that were subsequently verified by Sanger sequencing.

For the assessment of *EYA4* expression, we proceed similarly to our previous works^56^. COS7 cells were cultured in DMEM medium supplemented with 10% fetal calf serum (FCS), 100 U/ml of penicillin and 100 mg/ml of streptomycin on 12 mm glass coverslips (Menzel-Glaser, GmbH, Braunschweig, Germany) in flat-bottomed 24-well multititer plates (Falcon; BD Biosciences, San Jose, CA, USA) for immunocytochemistry studies. Cells were transiently transfected with 800 ng of the wild-type or with each of the two mutant constructs using Lipofectamine TM 2000 (111668, Invitrogen) according to the manufacturer’s directions. For immunocytochemistry, cells on coverslips were fixed 48 h after transfection in 4% paraformaldehyde in phosphate buffer (0.1 M NaH2PO4 pH 7.04) for 10 min and then washed three times briefly in PBS.

To study the intracellular protein localization of HA-tagged SIX1 and the c-myc tagged EYA4 proteins, cells were permeabilized for 10 min at room temperature with 0.5% Triton X-100 in PBS and blocked with 3% BSA in PBS for 30 min, washed twice with PBS and incubated for 1 h at room temperature with a 1:2000 and 1:1000 dilution of the rabbit primary antibody against the HA epitope (Abcam) and the mouse primary antibody against the Myc epitope (Sigma), respectively. Cells were then washed three times with PBS, and incubated for 1 h at room temperature with a 1:1000 dilution of the secondary antibody, Alexa FluorTM 488 conjugated goat anti-rabbit IgG (Molecular Probes A-11034; Invitrogen) or with the antibody Alexa 594 conjugated goat anti-mouse IgG (Molecular Probes A-11020; Invitrogen). After washing three times in PBS, slides were incubated with Hoechst 1μg/ml (Sigma, Hoechst 33342) for 10 min and washed one time in PBS. Slides were mounted using Fluorsave^TM^Reagent (Calbiochem, USA)

### Western blot analysis

Transfected COS7 cells were homogenized in lysis buffer (150 mM NaCl, 50 mM Tris–Cl, pH 7.4, 5 mM EGTA, 5 mM EDTA, 1% Triton X-100, 25 lg/ml leupeptin, and 1 mM phenylmethylsulphonylfluoride) and maintained on ice for 30 min. After centrifugation at 10,000 g (4 °C) for 5 min, 100 μg of the supernatant was mixed 1:1 with SDS-PAGE sample buffer (50 mM Tris–Cl, pH 6.8, 50 mM DTT, 2% SDS, 10% glycerol, 5% βME). The proteins were separated in 10% SDS-PAGE gels and transferred electrophoretically to nitrocellulose membranes. After blocking with 5% skimmed dry milk in 1% TBS (pH 7.6)/0.1% Tween 20 for 1 h at room temperature, the blots were incubated with the primary rabbit anti-HA antibody (1:2000) (Abcam) for 1 hour. After washing, the first antibody was detected by a secondary sheep anti-rabbit IgG antibody (1:1000) conjugated with HorseRadish peroxidase (HRP, Amersham Biosciences) for 1 h at room temperature. Bands were visualized by the enhanced chemiluminiscence (ECL; Amersham Biosciences) and exposed to X-ray film. The blots were then stripped, blocked and incubated with the primary mouse anti-Myc antibody (1:1000) (Sigma) for 1 hour. Secondary goat anti-mouse IgG antibody conjugated with HRP 1:1000 (Amersham Biosciences) was incubated for 1 hour. Visualization was carried out as previously described^57^.

### RNA extraction and cDNA synthesis

Total RNA was extracted from transfected COS7 cells using QIAamp® RNA kit following the manufacturer’s instructions (QIAGEN). Reverse transcription was carried out using 1μg of RNA and random hexamers with the Transcriptor First Strand cDNA Synthesis Kit (Roche). *EYA4* and Zeocin cDNAs were amplified using the primers *EYA4*F 5′-CGAGGAAGAGGCCGGAAA-3′ and *EYA4*R 5′-CAGCTTCCTCATCCAGTCCAC-3′ and ZeoF 5′-GGCTGCTCGCCGATCTCG-3′ and ZeoR 5-′GACCGGCTCGGGTTCTCC-3′ respectively and the 390 and 230 pb generated products were separated in a 2% agarose gel.

### Construction of splicing minigene and expression

Exons 12, 14, 15–16 and 17 of the *EYA4* human gene and its flanking introns were amplified by PCR using genomic DNA from controls and patients. PCR fragments were cloned into the pSPL3 exon trapping vector (Gibco BRL) digested with *XhoI* and *PstI* within the multiple cloning site. The pSPL3 vector^58^ contains an HIV genomic fragment with truncated *tat* exons 2 and 3 inserted into rabbit β-globin coding sequences. NIH3T3 cells were transfected with 4μg of the DNA plasmid using lipofectamin transfection reagent (Invitrogene). Cells were harvested 24 h post- transfection.

First strand cDNA was synthesized from 1 μg of total RNA by random-primed reverse transcription with the Transcriptor First Strand cDNA Synthesis Kit (Roche). To evaluate the transcriptional splicing pattern from the transfected minigenes, we used the forward primer SA (5′TCTGAGTCACCTGGACAACC-3′) and the reverse SD primer (5′-ATCTCAGTGGTATTTGTGAGC-3′), both of which annealed to the pSPL3 vector sequence. All spliced products were verified by Sanger sequencing.

## Results

### Identification of nine novel variants in *EYA4* gene associated with DFNA10

**Figure 1:**
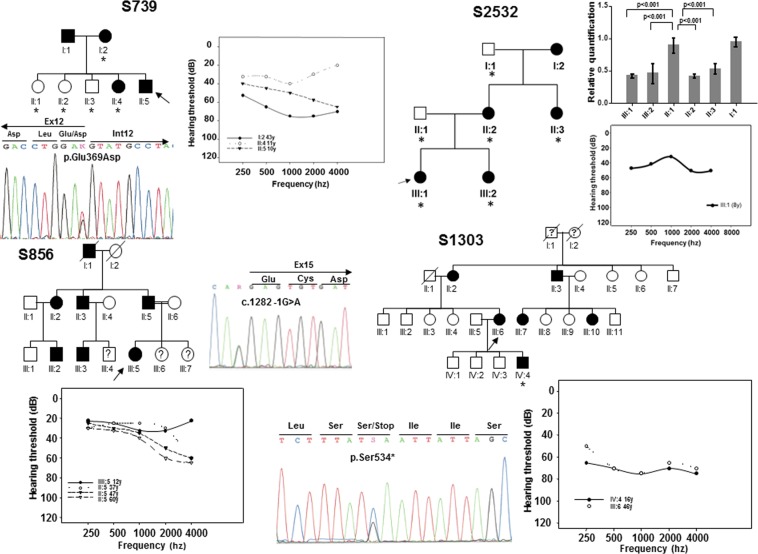
Pedigrees of the Spanish families S739, S856, S1303 and S2532. Black and white symbols indicate the affected and the unaffected subjects, respectively. The index cases are pointed by black arrows. The subjects of whom DNA samples were available for segregation analysis were marked by asterisks. The relative quantification of the 2747 bp deletion (CNV) by Real-Time PCR in the subjects of family S2532 is displayed. Only affected members showed a significant reduction (~50%) in the number of copies of *EYA4*. The audiograms showing the air conduction values obtained from several different patients of each family and the electropherograms of the mutations are also displayed. Each graph point represents the average hearing loss for the right and left ears.

**Figure 2:**
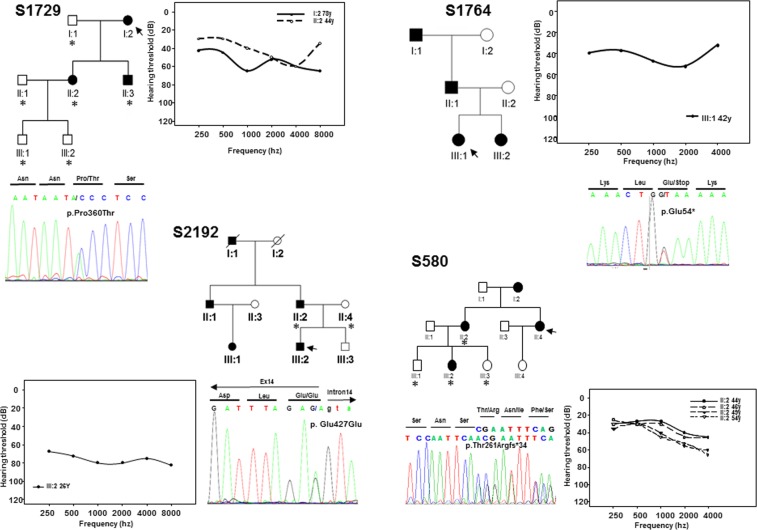
Pedigrees of the Spanish families S580, S1729, S1764, S2192 showing the audiograms and electropherograms as in Fig. 1.

In this study, we have enrolled, without phenotype-guided preselection, a cohort of 531 unrelated probands from Spanish families with non-syndromic SNHL in which the mode of inheritance was compatible with an autosomal dominant pattern. A first set of 312 patients were subjected to mutation screening by DHPLC enabling the detection of three novel variants in *EYA4* (Table 1). The first variant was identified in subject II:5 of family S739. It is a c.1107G > T transversion in exon 12 (Fig. 1) that replaces a glutamic acid at position 369 by aspartate (p.Glu369Asp). The second variant, c.1282-1G > A, was identified in subject III:5 of family S856 (Fig. 1) and affects the canonical 3′ acceptor splice-site of intron 14. The third nucleotide variation was the c.1601C > G transversion in exon 17 of *EYA4* in subject III:6 of family S1303 (Fig. 1) that produces a termination codon predicted to generate a truncated protein of 534 amino acids (p.Ser534*). Two extra subsets of 111 and 108 Spanish patients with ADSNHL were screened using OtoNGSpanel versions 1 and 2, respectively. Five extra novel pathogenic variants were identified using this methodology. A heterozygous deletion of 2747 bp in the index case (III:1) of family S2532 representing a copy variant loss encompassing exon 15 to exon 17 (Fig. 1). A stop mutation (p.Glu54*) in exon 4 in the index case (III:1) of family S1764 (Fig. 2); a frameshift deletion, c.781del in exon 10 detected in the index case (II:4) of family S580 predicted to create an aberrant protein tail of 34 amino acids and a premature stop codon (p.Thr261Argfs*34) (Fig. 2); a missense variant, c.1078C > A (p.Pro360Thr) in the index case (I:2) of family S1729 (Fig. 2), and a silent synonymous mutation, c.1281G > A (p.Glu427Glu) at the last nucleotide of exon 14 in subject III:2 of family S2192 (Fig. 2).

**Table 1:** All known *EYA4* (DFNA10) mutations including those identified in this study in boldface.

DNA change	Potein Change	Exon	Origin	Phenotype	Detection	Degree	Audiogram profile	Reference
c.84-2A > G		Intron 3	Chinese	SNHL	N.A	N.A	N.A	Chen *et al*. 2016^40^
c.152C > T	p.Ser51Phe	4	N. American	SNHL	N.A	N.A	N.A	Sloan-Heggen *et al*. 2016^29^
**c.160G** > **T**	p.Glu54*	**4**	**Spanish**	**SNHL**	**42 y**	**Mild**	**Mid/flat freq**.	***This work***
c.222_223del	p.Val75Phefs*32	5	Japanese	SNHL	61 y	Mild to moderate	High/Low freq.	Shinagawa *et al*. 2020^15^
c.464del	p.Pro155Glnfs*43 #	8	Swedish Dutch	SNHL SNHL	N.A Childhood	N.A Moderate	N.A Mid or high freq.	Neveling *et al*. 2013^16^ Van Beelen *et al*. 2016^17^
c.498del	p.Thr167Leufs*31	8	Japanese	SNHL	13	Mild	Low freq.	Shinagawa *et al*. 2020^15^
c.511G > C	p.Gly171Arg	8	Chinese	SNHL	2nd decade	Moderate to severe	Gently sloping	Liu *et al*. 2015^30^
c.517C > T	p.Gln173*	8	Japanese	SNHL	48 y	Moderate	Flat	Shinagawa *et al*. 2020^15^
c.579_580insTACC	p.Asp194Tyrfs*52	8	Swedish	SNHL	4–40 y	Mild to profound	Variable	Frykholm *et al*., 2015^18^
c.580 + 1G > A		Intron 8	Japanese	SNHL	45 y	Moderate	Flat	Shinagawa *et al*. 2020^15^
c.612dup	p.Glu205Argfs*40	9	Chinese	SNHL	20–40 y	Moderate to profound	High/flat freq.	Huang *et al*. 2015^19^
**c.781del**	p.Thr261Argfs*34	**10**	**Spanish**	**SNHL**	**26–44 y**	**Mild to moderate**	**Gently downsloping**	***This work***
c.804G > C	p.Gln268His	10	Slovak	SNHL	10–40 y	Moderate	Gently downsloping	Varga *et al*. 2019^31^
c.863C > A	p.Ser288*	11	Korean Korean	SNHL SNHL	N.A N.A	Moderate Moderate to severe	Reverse U-shaped Flat freq.	Baek *et al*. 2012^25^ Kim *et al*. 2015^26^
c.866C > T	p.Thr289Met	11	N. American	SNHL	N.A	N.A	N.A	Miszalski-Jamka *et al*. 2017^32^
c.910del	p.Ser305Leufs*15	11	Japanese	SNHL	30	Severe	Flat	Shinagawa *et al*. 2020^15^
c. 978C > G	p.Phe326Leu	12	Korean	SNHL	N.A	Moderate	Down sloping	Choi *et al*. 2013^33^
c.988C > T	p.Gln330*	12	Japanese	SNHL	16 y	Moderate	Flat	Shinagawa *et al*. 2020^15^
c.1026_1027dup	p.Thr343Lysfs*62	12	N. American	SNHL	1st, 3rd decade	Moderate to profound	Flat/Gently sloping	Wayne *et al*. 2001^20^
c.1048_1049dup	p.Arg352Profs*53	12	N. American	SNHL	2nd-4th decade	Moderate to severe	Mid to high freq.	Makishima *et al*. 2007^21^
**c.1078C** > **A**	**p.Pro360Thr**	**12**	**Spanish**	**SNHL**	**44 y**	**Mild to moderate**	**Gently downsloping**	***This work***
**c.1107G** > **T**	**p.Glu369Asp**	**12**	**Spanish**	**SNHL**	**10–11 y**	**Mild to severe**	**Gently downsloping**	***This work***
c.1109G > A	p.Arg370His	13	Philippines	SNHL	N.A	N.A.	N.A	Truong *et al*. 2019^34^
c.1109G > C	p.Arg370Pro	13	Japanese	SNHL	30 y	Mild to moderate	Mid freq.	Shinagawa *et al*. 2020^15^
c.1111G > A	p.Val371Met	13	Belgium	SNHL	N.A	N.A	N.A	Sommen *et al*. 2016^35^
c.1115_1118dup	p.Trp374Cysfs*6	13	Hungarian	SNHL	Postlingual	Profound	Variable	Pfister *et al*. 2002^22^
**c.1122G** > **T**	**p.Trp374Cys**	**13**	**Australian**	**SNHL**	**10–25 y**	**Mild to severe**	**Gently downsloping**	***This work***
c.1154C > T	p.Ser385Leu	13	Italian	SNHL	Postlingual	Mild to profound	Mid-freq.	Cesca *et al*. 2018^36^
c.1177C > T	p.Gln393*	13	Korean Japanese Japanese	SNHL SNHL + minor DCM SNHL	N.A 26 y 26 y	Moderate to profound Moderate Moderate	Mid/High freq. Mid/flat freq. Flat	Kim *et al*. 2015^26^ Abe *et al*. 2018^27^ Shinagawa *et al*. 2020^15^
c.1194del	p.Met401Trpfs*3	14	Korean	SNHL	1st decade	Moderate	Down sloping	Choi *et al*. 2016^23^
c.1216G > C	p.Gly406Arg	14	Japanese	SNHL	5 y	Moderate	Flat	Shinagawa *et al*. 2020^15^
c.1223G > A	p.Arg408His	14	N. America	SNHL	N.A	N.A	N.A	Miszalski-Jamka *et al*. 2017^32^
**c.1281G** > **A**	**p.Glu427Glu**	**14**	**Spanish**	**SNHL**	**26 y**	**Moderate to profound**	**Flat freq**.	***This work***
c.1282 -12T > A		Intron 14	Australian	SNHL	1st-4th decade	Mild to profound	Mid/flat freq.	Hildebrand *et al*. 2007^41^
**c.1282 -1G** > **A**	**—**	**Intron 14**	**Spanish**	**SNHL**	**12 y**	**Mild to moderate**	**Gently downsloping**	***This work***
c.1301T > A	p.Ile434Lys	15	Chinese	SNHL	8-38 y	Mild to severe	Mid/flat freq.	Tan *et al*. 2014^37^
c.1341 -19T > A		Intron 15	Germany	SNHL	N.A	N.A	N.A	Vona *et al*. 2014^42^
**c.1601C** > **G**	p.Ser534*	**17**	**Spanish**	**SNHL**	**3–16 y**	**Moderate to severe**	**Mid/flat freq**.	***This work***
c.1643C > G	p.Thr548Arg	18	Chinese	SNHL	17–40 y	Mild to profound	Variable	Sun *et al*. 2015^38^
c.1663G > C	p.Ala555Pro	18	Japanese	SNHL	25 y	Moderate	High freq.	Shinagawa *et al*. 2020^15^
c.1739 -1G > A		Intron 18	N. America	SNHL	50 y	N.A	N.A	Cirino *et al*. 2017^43^
c.1759C > T	p.Arg587*	19	Belgian	SNHL	6–40 y	Mild to moderate	Mid freq.	Wayne *et al*. 2001^20^
c.1790del	p.Val597Glyfs*4	19	Japanese	SNHL	35	Moderate	Flat	Iwasa *et al*. 2016^24^
c.1810G > T	p.Gly604Cys #	19	Swedish Dutch	SNHL	N.A	N.A	N.A	Neveling *et al*. 2013^16^ Van Beelen *et al*. 2016^17^
c.1834A > T	p.Lys612*	19	Chinese	SNHL	27 y	Moderate	Gently downsloping	Hu *et al*. 2018^28^
c.1855T > G	p.Trp619Gly	20	Chinese	SNHL	N.A	N.A	N.A	Xiao *et al*. 2019^39^
Deletion 7689 bp (Ex7 to Ex11)			Japanese	SNHL	25 y	Moderate to severe	Low/High freq.	Shinagawa *et al*. 2020^15^
Deletion 9.5 Mb (Ex4 to Ex 20)			Japanese	SNHL	13 y	Severe	Low/High freq.	Shinagawa *et al*. 2020^15^
**Deletion 2747 bp (Ex15 to Ex17)**			**Spanish**	**SNHL**	**8 y**	**Moderate**	**Flat**	***This work***
Deletion 9 Mb at 6q23.1-24.1	No protein	-	Polish	SNHL +DCM + MR	N.A	N.A	N.A	Dutrannoy *et al*. 2009^45^
Deletion 4,846pb incl. intron 9, exon 10 and partial intron 10 c.581_804del	p.Asp194Glyfs*30	9–10	N.A	SNHL + DCM	2nd decade	Moderate to severe	Mid/flat freq.	Schönberger *et al*. 2000;^46^ 2005^7^
~10.4 Mb, incl. promoter and ex. 1-2 at 6q22.31-q23.2	—	—	Japanese	MR	20-months-old	N.A	N.A	Abe *et al*. 2009^47^
Deletion 3.7MB in 6q23.1q23.2	—	—	Italian	OTFCS + SNHL	12 y (SNHL)	Mild	N.A	Gana *et al*. 2019^48^

*EYA4* mRNA and protein sequences are: *NM_004100.5* and *NP_004091.3*. Nucleotide numbering reflects cDNA coordinates with +1 corresponding to the A of the ATG initiation codon of the CDS. SNHL sensorineural hearing loss, DCM autosomal dominant dilated cardiomyopathy, MR mental retardation, OTFCS otofaciocervical syndrome, NA not available. All variant names were checked using Mutalyzer 2.0.beta-21 software^59^. ^#^These mutations were found in the same patient.

**Figure 3:**
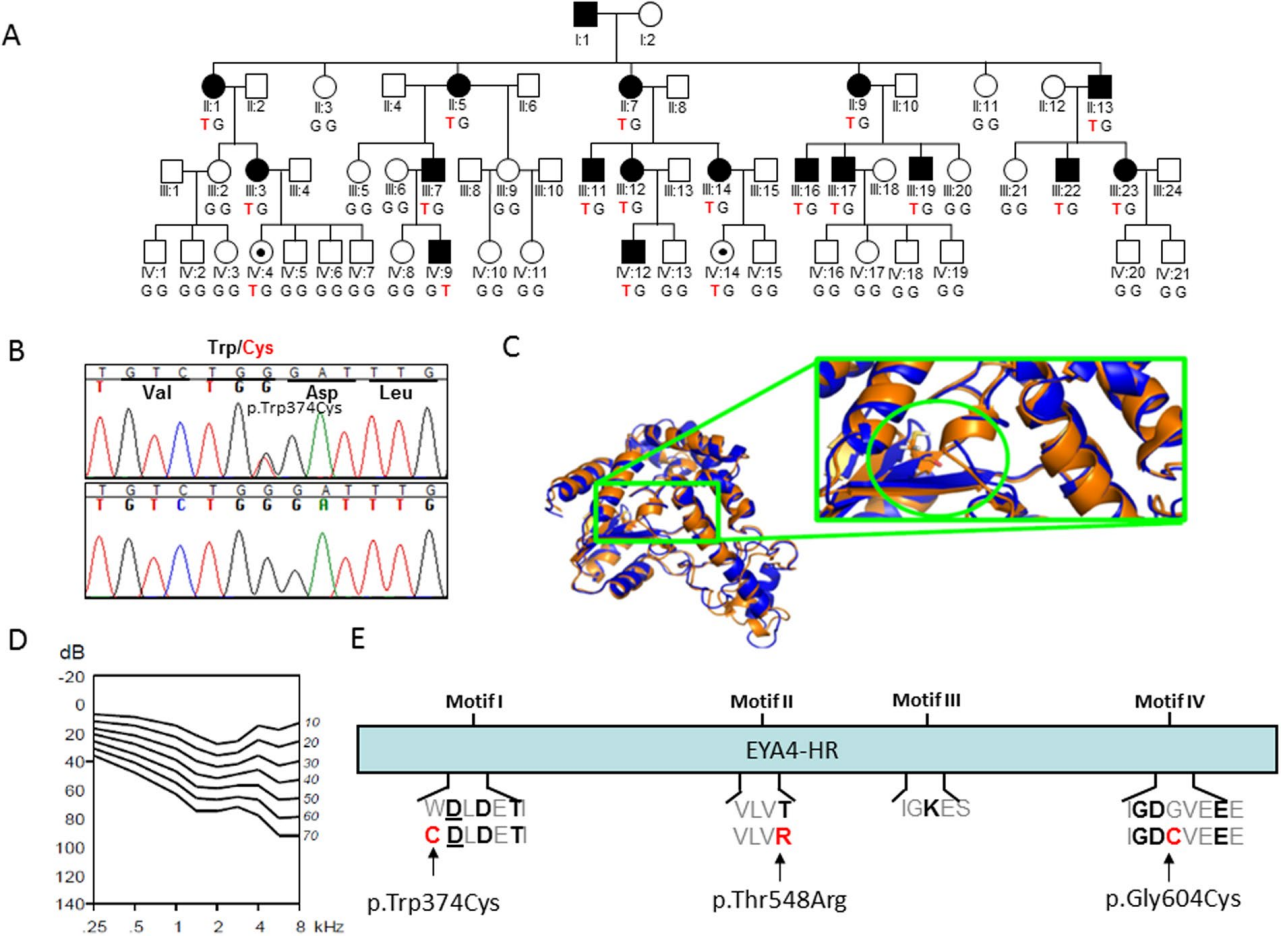
(**A**) Pedigree of the family 10880 showing the novel *EYA4* (c.1122G > T) variant segregating with the hearing loss phenotype. The genotype of all the subjects analysed is indicated in capital letters (Wt = GG, Hz = GT). Black dots represent normal hearing individuals carrying the mutation but under the age of onset in this family. (**B)** Representative chromatograms from wild-type and mutant sequences. (**C)** Molecular modelling of the p.Trp374Cys in EYA4. Mutated site is highlighted by a green circle and locally zoomed. Mutant (orange) is superimposed on wild-type (blue) for comparison. The p.Trp374Cys alters local conformation and disrupts beta sheet backbone folding resulting in decrease structural stability. (**D)** Age Related Typical Audiogram (ARTA) analysis. The hearing loss progresses at a rate that ranges between 0.5 dB/year at 0.25Khz and 1.3 dB/year at 8 kHz. **E)** EYA4-HR domain showing the four Tyr-phosphatase motifs that mediate the protein enzymatic activity. The p.Trp374Cys mutation identified in this work, and two previously identified missense mutations (p.Thr548Arg and p.Gly604Cys) are all affecting conserved residues at motif I, II and IV, respectively.

Finally, a novel missense variant, c.1122G > T (p.Trp374Cys) was identified in the Australian family (10880) using the gene panel OtoSCOPE (Fig. 3). Segregation with the hearing loss for the different mutations was confirmed in all the extended families of whom DNA samples were available. The segregation of the CNV (deletion of exon 15 to 17) in members of family S2532 was assessed by relative quantification of the exon 16 by Real-time PCR (Fig. 1). The classification for each SNV was done taking into account the American College of Medical Genetics and Genomics (ACMG) guidelines and the prediction algorithms used by Varsome^60,61^, several predictors of pathogenicity, the Human Splice Finder (HSF) predictor^62^, the allele frequencies in the Genome Aggregation Database (GnomAD)^63^, the international genome sample resource (IGSR, https://www.internationalgenome.org/data), the Exome Aggregation Consortium-ExAC (http://exac.broadinstitute.org/), the Collaborative Spanish Variant Server-CSVS database (http://csvs.babelomics.org/) and the Deafness Variation Database (DVD, http://deafnessvariationdatabase.org/). This analysis indicated that six out of eight single nucleotide variants (SNVs) identified in this study were classified as “Pathogenic/Likely pathogenic”, whereas two remained as “variants of uncertain significance” (VUS) (Table 2).

**Table 2:** Classification of Single Nucleotide Variants (SNVs) identified in this work.

DNA change	Protein change	ACMG	Number of scores supporting pathogenicity	HSF	DVD	CSVS Allele Freq.	GnomAD Allele Freq.
c.160G > T	p.Glu54*	Pathogenic (PVS1, PM2, PP1, PP3)	6/9	Activation of an exonic cryptic donor site. Potential alteration of splicing. Alteration of an exonic ESE site. Potential alteration of splicing.	N.A	0	0
c.781del	p.Thr261Argfs*34	Pathogenic (PVS1, PM2, PP1, PP3)	N.A	Alteration of an exonic ESE site. Potential alteration of splicing.	N.A	0	0
c.1078C > A	p.Pro360Thr	VUS (PM2, PP1, PP3, BP1)	16/19	No impact on splicing	VUS	0	1.06e-5 (3 in 281904)
c.1107G > T	p.Glu369Asp	Likely pathogenic (PS3, PM2, PP1, PP3, BP1)	11/20	Alteration of Wt donor site. Alteration of an exonic ESE site	N.A	0	0
c.1122G > T	p.Trp374Cys	VUS (PM2, PP1, PP3, BP1)	19/19	Activation of an exonic cryptic donor site.	N.A	0	0
c.1281G > A	p.Glu427Glu	Likely pathogenic (PS3, PM1, PM2, PP1, BP4)	1/1	Alteration of Wt donor site, most probably affecting splicing	N.A	0	0
c.1282-1G > A	—	Pathogenic (PVS1, PS3, PM2, PP1, PP3)	6/6	Alteration of the Wt acceptor site, most probably affecting splicing. Activation of an intronic cryptic acceptor site. Potential alteration of splicing.	N.A	0	0
c.1601C > G	p.Ser534*	Pathogenic (PVS1, PS3, PM1, PM2, PP1, PP3)	7/9	Creation of an exonic ESS site	N.A	0	0

ACMG criteria: **PVS1** (**Pathogenic Very Strong**): null variant (nonsense, frameshift, canonical ±1 or 2 splice sites, initiation codon, single or multiexon deletion) in a gene where LOF is a known mechanism of disease. **PS3** (**Pathogenic Strong 3**): well-established *in vitro* or *in vivo* functional studies supportive of a damaging effect on the gene or gene product. **PM1** (**Pathogenic Moderate 1**): located in a mutational hot spot and/or critical and well-established functional domain (e.g., active site of an enzyme) without benign variation. **PM2** (**Pathogenic Moderate 2**): absent from controls (or at extremely low frequency if recessive) in Exome Sequencing Project, 1000 Genomes Project, or Exome Aggregation Consortium. **PP1** (**Pathogenic Supporting** 1): cosegregation with disease in multiple affected family members in a gene definitively known to cause the disease. **PP3** (**Pathogenic Supporting** **3**): multiple lines of computational evidence support a deleterious effect on the gene or gene product (conservation, evolutionary, splicing impact, etc.). **BP1** (**Benign Supporting** **1**): missense variant in a gene for which primarily truncating variants are known to cause disease. **BP4** (**Benign Supporting** **2**): multiple lines of computational evidence suggest no impact on gene or gene product (conservation, evolutionary, splicing impact, etc.). All the databases were searched on the 22nd of March 2020.

**Figure 4:**
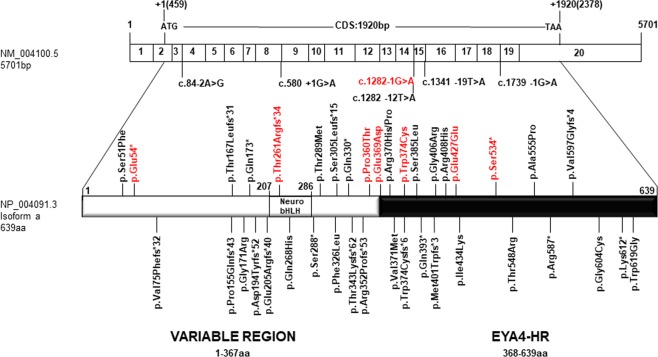
Scheme depicting the mRNA structure and coding sequence (CDS) of the NM_004100.5 *EYA4* transcript and the NP_004091.3 protein isoform showing the variable region and the EYA4 homologous region (EYA4-HR domain). The novel genetics variants (in red) and those previously identified in *EYA4* are shown.

All mutations identified so far in *EYA4* gene, including those reported in the present work, are summarized in Table 1 and Fig. 4. The hearing loss observed in our patients with *EYA4* mutations is characteristic of the DFNA10 phenotype. Affected subject show a bilateral and progressive hearing loss of postlingual onset (ranging from the first to fourth decade) with a flat/gently downsloping audiometric profile (Figs. 1, 2). The ARTA of the Australian family (10880) provided evidence that the hearing loss progresses at a rate that ranges between 0.5 dB/year at 0.25 kHz and 1.3 dB/year at 8 kHz (Fig. 3D).

### Effect of p.Glu369Asp and p.Ser534* mutations on EYA4 localization

**Figure 5:**
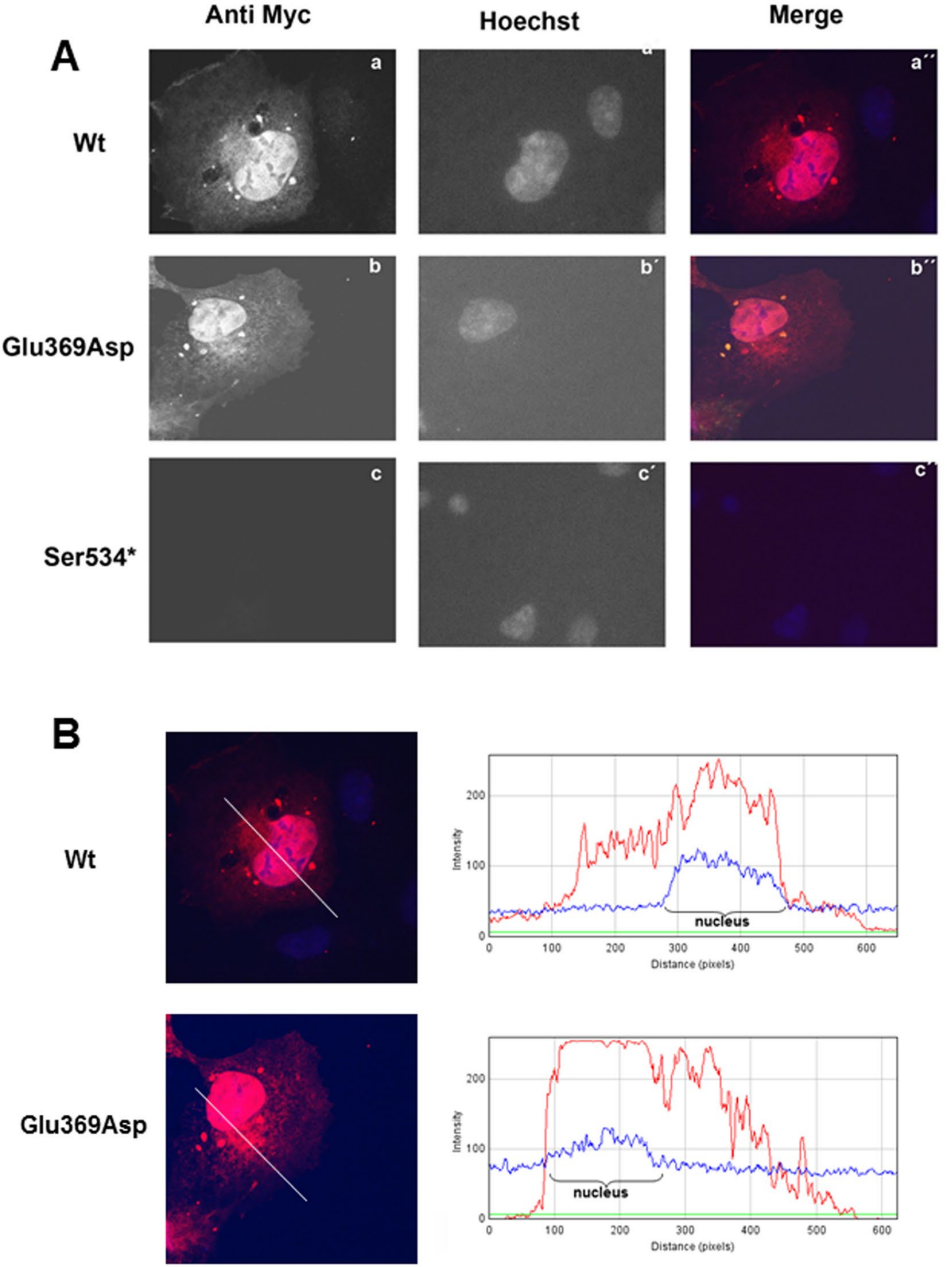
(**A**) Expression pattern of the EYA4-HR Wt (a), Glu369Asp (b) and Ser534* (c) in COS7 cells. EYA4-HR protein shows a cytoplasmic and nuclear distribution for the Wt and Glu369Asp mutant but no signal was observed for the mutant Ser534*. Hoechst staining was used to identify the nucleus. (**B)** IMAGE J graphical section showing the fluorescence intensities plotted against the distances (pixels). EYA4-HR staining (in red) for the Wt and Glu369Asp mutant is detected in both the nucleus and the cytoplasm.

**Figure 6:**
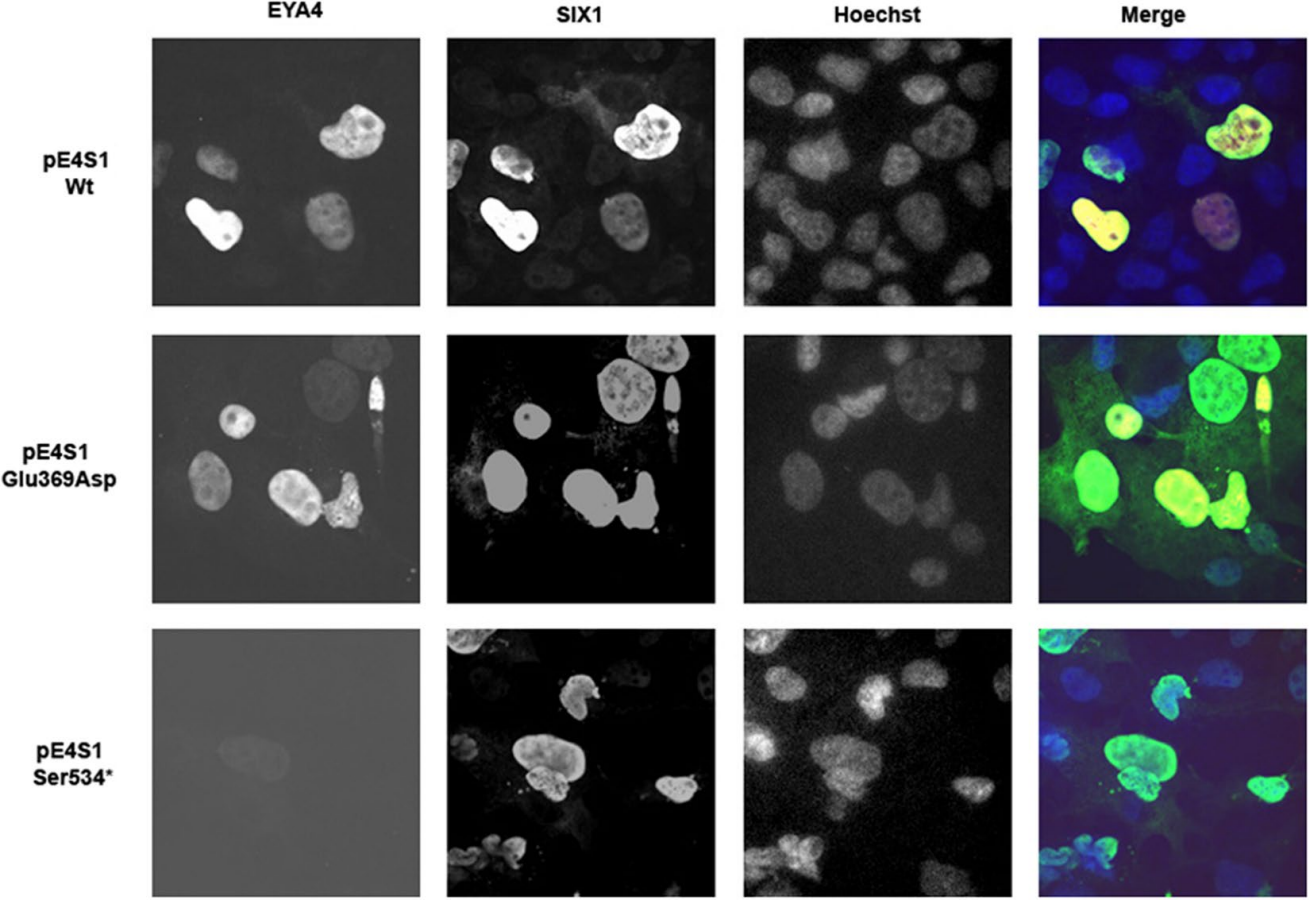
Nuclear translocation of the EYA4-HR-SIX1 Wt and mutant protein complex in COS 7 cells. In presence of SIX1, EYA4-HR Wt and Glu369Asp were detected mostly in the nucleus, consequently, the capacity of nuclear translocation of the complex EYA4-SIX1 seems not to be impaired by the p.Glu369Asp mutation. No signal was observed for p.Ser534* mutant.

To further explore the mechanism of pathogenesis underlying p.Glu369Asp and p.Ser534* mutations, we investigated their effect on interaction with SIX1 and ability of the SIX1-EYA4 protein complex to translocate to the cell nucleus. To do this we investigated the expression pattern and cellular localization of EYA4 mutants in transiently transfected COS7 cells in the absence or presence of SIX1. When SIX1 protein is not present, the c-myc-tagged EYA4-HR-Wt and the EYA4-HR-Glu369Asp proteins are detected in both, cytoplasm and nucleus, in the majority of COS7 cells forty-eight hours after transfection (Fig. 5A,B). However, no signal was observed for the mutant p.Ser534* (Fig. 5A). When COS7 cells were transfected with the bicistronic plasmid containing SIX1 and EYA4-HR (pS1E4), the protein complex was detected mostly in the nucleus for both Wt and p.Glu369Asp mutant, but not for p.Ser534* mutant (Fig. 6; Supp Fig. 2A,B). Taken together, these observations suggest that p.Glu369Asp mutation does not significantly alter the interaction with SIX1 and consequently does not impair the nuclear translocation of the EYA4-SIX1 complex.

**Figure 7:**
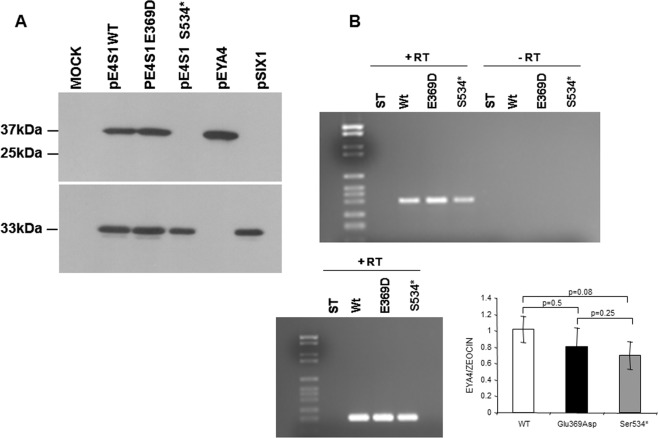
(**A**) Western blot analysis of EYA4 Wt and mutants’ production. A clear band of 37 kDa corresponding to the EYA4-HR domain detected by the anti-myc antibodies directed against the c-myc tag cloned in frame in the Nt portion of the EYA4-HR domain (Suppl Fig. 1) is observed in extracts from COS7 cells transfected with pE4S1 Wt and Glu369Asp bicistronic plasmid and in the control p*EYA4*. No signal was detected in the mutant Ser534* when we use the same anti-Myc antibodies (at the top), whereas a robust band of 33kDA corresponding to SIX1 was seen when we use anti-HA antibodies (at the bottom) to detect the HA-tagged SIX1 protein (Suppl Fig. 1). The full-length blots revealed at different time exposures with anti-Myc and anti-HA antibodies are displayed in Suppl Fig. 5. (**B) (top)** RT-PCR assay performed on total RNA extracted from COS7 cells transfected with Eya constructs (wild-type and mutants) showed a band corresponding to the amplification of the tagged EYA4-HR mRNA. This band was not detected in the control lanes (-RT) in which the mRNA was nod added; (**B) (bottom)** cDNA amplification of Zeocin (the plasmid antibiotic resistant cassette) using specific primer was used for normalization purposes. Quantification of the relative amounts of *EYA4* cDNA amplifications once they were normalized with Zeocin levels did not show any statistically significant differences after Wt and mutants comparisons. t Student p values are shown.

To confirm the results of the immunocytochemistry assays, we performed a western-blot analysis of COS7 cells transfected with the pE4S1Wt, pE4S1Glu369Asp and pE4S1Ser534* vectors. Lysates were separated on a 10% gel followed by blotting and immunostaining with the anti-myc and anti-HA antibodies. As shown in Fig. 7A a clear band of 37 kDa corresponding to the EYA4-HR domain was detected for the Wt and p.Glu369Asp mutant using anti-myc antibodies directed against the c-myc tag cloned in frame in the Nt portion of the EYA4-HR domain (Suppl Fig. 1). However, by using the same anti-myc antibodies the 25 kDa band expected for the putative truncated protein p.Ser534*, was not detected in the corresponding lane, whilst a clear band of 33 kDa was observed when anti-HA was used to detect the HA-tagged SIX1 protein (Suppl Fig. 1) indicating an efficient transfection with the bicistronic plasmid. Therefore, the p.Ser534* mutant protein could not be detected in transfected cells neither by immunocytochemistry nor by Western blotting analysis.

The lack of detection of p.Ser534* mutant could be explained by a degradation at the mRNA or protein levels. To test this hypothesis, we obtained total RNA from COS7 cell transfected with the Eya constructs (pE4-Wt, pE4_Glu369Asp_ and pE4_Ser534*_) from three independent experiments and RT-PCR assays were then carried out. Expected PCR-amplimers of 390 pb were obtained in the Wt and both mutants by using specific primers to amplify an EYAHR fragment. No statistical significant differences in mRNA expression levels were found between the wild type and mutant transcripts (Fig. 7B) once they were normalized with the expression level obtained from the Zeocin mRNA, the antibiotic selection marker of the vector.

### *In Silico* splicing analysis of *EYA4* mutations

To further investigate the pathogenic mechanism of p.Glu369Asp missense mutation we performed computational analysis of the genomic region surrounding c.1107G > T which corresponds to the last nucleotide of exon 12, a position important for normal splicing. We used several different web-based tools: Esefinder, MaxenScan, BDGP Splice Site Prediction by Neural Netwok and NetGene2^64–68^. Esefinder and Netgene2 predict a complete loss of the splicing donor site for the mutant transcript. MaxEntScan evaluates 5′ splice site strengths, being the typical MaxEnt 5′ score in wild type sequences of around 11.81. In our analysis MaxEntScan yielded a score of 9.12 for the Wt 5′splice site of intron 12 in *EYA4* and 3.89 for the c.1107G > T mutant. These data suggest that c.1107G > T alters the 5′ splice site leading to skipping of exon 12 and generation of a truncated protein of 331 amino acids. Similar analysis was performed for the c.1282-1G > A mutation identified in family S856. ESEfinder and MaxEntscan softwares indicated that c.1282-1G > A would abrogate the 3′ splicing acceptor site of intron 14 leading to skipping of exon 15. The aberrant splicing of exon 15 would produce a putative truncated protein of 433 amino acids or, as an alternative, a peptide of 455 amino acids if the cryptic splice acceptor site within the exon 16 was used, as previously described^4^. The same analysis was performed for the nonsense mutation (c.1601C > G, p.Ser534*) and for the other missense mutations identified in this study (p.Pro360Thr; p.Trp374Cys) but no alteration of splicing was predicted. Finally, we analyzed the silent mutation c.1281G > A (p.Glu427Glu) as it affects the last nucleotide of exon 14 and the *in silico* analysis carried out by ESEfinder and MaxEntscan software predicted this mutation could be compromising the splicing donor site of intron 14 (Table 2).

### *In vivo* minigene splicing assays of *EYA4* mutations

**Figure 8:**
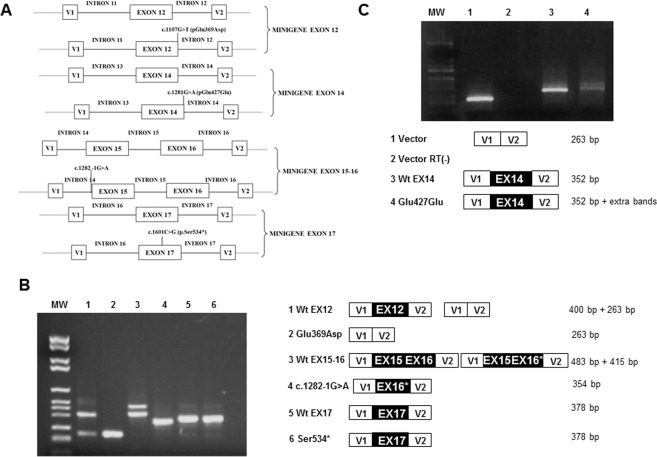
Minigene assays. (**A)** Scheme of the different minigene constructs for the exon 12, 14, 15–16 and exon 17 genomic regions. (**B,C)** PCR products of the minigene constructs obtained using SA and SD primers inside pSPL3 vector. MW: DNA Molecular Weight VI (Roche). V1 and V2 denote the artificial exons of the pSPL3 vector. The sizes of the PCR products are shown on the right. In B) lanes 1 and 2 show the PCR products of the constructs carrying exon 12 wild type (lane 1) and c.1107G > T (Glu369Asp) (lane 2), exon 15–16 wild type (lane 3), c.1282 -1G > A (lane 4), exon 17 wild type (lane 5) and c.1601C > G (Ser534*)(lane 6). Ex12, Ex15, Ex16, Ex17 and Ex16* of *EYA4* gene denote exons 12, 15, 16, 17 and 16 lacking the first 68 pb, respectively. In C) lanes 1 and 2 show the PCR products of the empty vector (263 bp) and the RT negative control, respectively. Lane 3 shows a dramatic reduction in the intensity of the 352pb band and the presence of supernumerary bands.

To assess the predicted effects of *EYA4* mutations on splicing, we carried out minigene assays in NIH3T3 cells (Fig. 8A). Results of the minigene indicated that the wild type exon 12 was correctly incorporated in more than 85% of the transcripts (400 bp band), while the mutant c.1107G > T (p.Glu369Asp) failed to be retained in the mature transcript and was eliminated during the splicing process (263 bp band). These results confirm the *in silico* analysis indicating that this mutation compromises the acceptor splicing site of exon 12. This would result in a frameshift and a premature termination codon generating a truncated *EYA4* protein (Fig. 8B, Supp Fig. 3A).

The wild type minigene including exons 15–16 generated two splicing products, a 483 bp band that matched with the expected size for the normal splicing of exon 15 and 16 and a 415 bp band corresponding to normal exon 15 and a shorter exon 16 that lacks the first 68 nucleotides (Fig. 8B, Supp Fig. 3B). The shorter exon 16 was previously identified in human lymphoblastoid and lens epithelial cell lines^4^. The c.1282-1G > A mutant minigene showed a PCR product of 354 nucleotides that lacks exon 15 and includes the shorter exon 16 (Fig. 8B, Supp Fig. 3B).

Results of the minigene assays for the Wt and c.1601C > G (p.Ser534*) mutant in exon 17, revealed a single 378 bp PCR product corresponding to the mature transcript in which the exon 17 is retained. These data indicate that this mutation has no effects on splicing as the *in silico* analysis had previously indicated (Fig. 8B, Supp Fig. 3C).

Finally, the minigene construct containing both, the Wt and c.1281G > A (p.Glu427Glu) mutant in exon 14, revealed that wild type exon 14 is incorporated in more than 90% of the transcripts (352 bp band). However, the intensity of this band was dramatically reduced in the mutant. Additionally, extra bands were also observed, indicating an impairment of the splicing process (Fig. 8C).

## Discussion

Transcription factors and co-factors are large group proteins that are fundamental for proper gene regulation. These factors often exhibit a combination of spatial and temporal expression and are critical for cellular events including development, function and lifelong maintenance. In the inner ear, disruption of transcription factors belonging to the POU, SIX, LMX and EYA families have all been shown to be essential for proper hearing in humans. Here we report 9 novel mutations that disrupt the expression or function of the *EYA4* gene, segregating in 8 Spanish families and one Australian family. In doing so we report the first synonymous variant, classified as likely pathogenic following the ACMG criteria and the minigene assay, linked to DFNA10 and provide further evidence for haploinsufficiency as the common underlying disease-causing mechanism for DFNA10-related hearing loss.

In previous mutational screenings of ADNSHL families in the Spanish population, only 15 *TECTA* (DFNA8/12), two *ACTG1 (*DFNA20/26) and two *mi*R-96 (DFNA50) mutations were identified^54,56,69^. In this study, we have identified 8 novel variants in *EYA4* gene after mutational screening of 531 Spanish familial cases. Therefore, the global prevalence of DFNA10 in the Spanish hearing impaired is about 1.5% (8/531), suggesting that it is rare form of ADNSHL in Spain. Moreover, we have identified a novel missense mutation in *EYA4* in an Australian family enrolled in this study. The exact prevalence of *EYA4*/DFNA10-related hearing loss is not known as it was only detected in Caucasian and East Asian population to date. However, studies from Japan, Korea and Slovakia estimate the prevalence of *EYA4*-related hearing loss to be 0.9%^15^, 7.4% (1/14)^33^ and 5.56% (1/17)^31^. The substantial increased prevalence in the last two populations is most likely due to the reduced number of probands screened and the phenotypic preselection of patients.

Prior to this study, 47 families with ADSNHL at the DFNA10 locus have been identified. Despite the different mutations (nonsense, missense, splice-site, frameshift or large genomic deletions) so far reported in the DFNA10 families and the different affected protein domains in EYA4, no dramatic auditory phenotypic differences have been observed. To the best of our knowledge, all families carrying mutations in *EYA4* exhibit a similar phenotype consisting of a postlingual progressive hearing impairment affecting high frequencies at greater rates. ARTA analysis in DFNA10 families for which audiograms were available (primary or published data) demonstrates a fairly similar picture (Suppl Fig. 4). Relatively discordant phenotypes showing poorer thresholds, i.e. of> 45 dB at ages of> 20 years at all frequencies, were seen in a few patients who carried two mutations, p.Pro155Glnfs*43 and p.Gly604Cys^17^.

The ATD indicated more progression at high frequencies in most families. Progression varied from ~0.5 - ~0.8 dB/year at 0.25–1 kHz to ~1.5 - ~1.8 dB/year at 4–8 kHz. Exceptions were for LMG 265 with the p.Arg352Profs*53 mutation^21^ showing progression with an ATD of ~0.8 dB/year at all frequencies, and the Australian family harboring a splice-site mutation c.1282-12 T > A, which is predicted to result in exon 15 skipping^41^, that showed more progression at the lower frequencies (ATD ~1.5 dB/year at 0.25–1 kHz) than at the higher frequencies (ATD ~1.0 dB at 4–8 kHz).

Mutations in *EYA4* have also been linked to HL and dilated cardiomyopathy (DCM) in two families^7,45,46^. The genotype-phenotype correlation for DFNA10 *versus* HL with DCM is not well understood. Initially, it was proposed that mutations resulting in the loss of the EYA-domain would result in HL with DCM. However, this was later challenged as several families have been reported with truncating mutations in the variable region with no DCM phenotype (Table 1). In this work we reports the most N-terminal nonsense mutation identified to date in the variable region (c.160G > T; p.Glu54*) that is only associated with SNHL in a Spanish family. Interestingly, one mutation (p.Gln393*), has been linked to both DFNA10^15,26^ and HL with a mild-DCM^27^. The differences in the cardiovascular phenotype could be due to environmental factors, variable penetrance or a coincidental segregation of different genetic etiology for mild-DCM in the family described by Abe *et al*. (2018). In our study, all reported families have non-syndromic DFNA10-related hearing loss regardless of mutation type and location.

It is noteworthy that families with available clinical data and large deletions encompassing either the whole gene or part of the gene, have other clinical phenotypes which include, dilated cardiomyopathy (DCM) and/or isolated mental retardation and OTFC syndrome^7,45,47,48^. It is unclear whether these additional phenotypes are a result of contiguous gene deletions or more complex, mechanisms are at play. For example, the c.579_580insTACC, p.Asp194Tyrfs*52; mutation associated with SNHL^18^ is indeed very similar to the truncated isoform (c.581_804del; p.Asp194Glyfs*30) resulting from the 4,846pb DNA deletion spanning the intron 9-exon 10-partial intron 10 described in the family with SNHL and dilated cardiomyopathy^7,46^. Moreover, we have identified the first gross heterozygous deletion reported in an Spanish family spanning exon 15 to 17 that is only associated with SNHL, and a recent work^15^ also reports two more CNVs (affecting exon 7 to 11 and exon 4 to 20, respectively) linked to SNHL. Thus, one possibility is that the reported cardiomyopathy in other families was caused by other genetic factors present in the large deleted region or created by the event of deletion.

Prior to this study, 16 out of 38 variations identified in *EYA4* are missense mutations (Table 1). The variant p.Trp374Cys identified in this work causes a substitution from a large, bulky and hydrophobic tryptophan to a small thiol-containing cystine at amino acid 374. We predict this change might alter protein folding or conformation, possibly by allowing the thiol of the substituted cystine to create new disulfide bonds and disrupting proper protein folding. Improper protein folding or formation may interfere with its other protein binding partners and an inability to properly form the essential PSED network^7,20,44^. An alternative hypothesis would be a detrimental effect on the activation and enzymatic activity of EYA4 by modifying metal cofactor binding ability of magnesium ions (Mg^2+^). There are three predicted metal binding sites (amino acids 375, 377 and 603) and two active sites (amino acids 375 and 377) in the EYA domain (Fig. 3C,E). Interestingly, all three of these residues are Aspartic Acid and are all integral parts of catalytic motifs fundamental to EYA4’s Tyr phosphatase activity. Unlike traditional Tyr phosphatases which require Cystine residues at the heart of their catalytic core, EYA proteins depend on Aspartic Acid residues and require divalent ion, such as Mg^2+^ for activation^6^. The first motif (AA sequence [WDLDETI]) starts at residue 374 and extends 6 residues to 380 (Fig. 3E). The Aspartic Acid at position 375 acts as the catalytic center and is essential for EYA4 activation. It is possible that this variant does not cause a dysregulation of the PSEDN, but rather alters EYA4’s enzymic activity, either physically (changing the protein conformation of the binding pocket) or chemically (altering binding affinity and efficiency of its neighboring Aspartic Acids through the cystine thiol-group known to have a high affinity for other metals)^70^ or a combination of both. This possible activation impairment could adversely affect the enzymic roles of EYA4 in the inner ear. In any case, more functional studies are required to fully assess the consequences of this variant. Interestingly, a variant has already been reported at this amino acid position, (p.Trp374Cysfs*6) in a Hungarian family with progressive ADNSHL^22^. The reported family exhibits a similar phenotype as family 10880. Surprisingly, mutation type and location do not seem to influence the hearing phenotype as seen in other deafness-causing genes^54^.

The variant c.1078C > A; p.Pro360Thr was not present in the disease or hearing-loss genetic variant databases consulted and it presented an extremely low allelic frequency in the GnomAD. It causes the substitution from the non-polar, aliphatic residue proline to a polar non-charged residue threonine at position 360. We predict this change might alter protein folding or conformation, as the distinctive cyclic structure of proline’s side chain gives proline an exceptional conformational rigidity compared to other amino acids. This proline is fully conserved through evolution and the computational analysis (16 out of 19, including Polyphen-2, Sift and others) indicated the p.Pro360Thr is a damaging change. However, in the absence of functional analysis it is classified as a VUS following the ACMG criteria.

The p.Glu369Asp mutation identified in this work initially categorized as a missense mutation does not affect a key position for EYA4 activation or enzymatic activity. Furthermore, it behaved as the wild type isoform in the *in vivo* experiments we have carried out as it did not impact the translocation of the mutant SIX1-EYA4Glu369Asp complex to the nucleus. Both *in silico* predictions and minigene assays, confirmed that the c.1107G > T (pGlu369Asp) affects the donor splice-site of exon 12. The human 5′ss consensus sequence is MAG|GURAGU (M is A or C; R is purine), spans positions −3 to +6 relative to the exon-intron junction^71^. Pathogenic splicing alterations caused by point mutations in both 5′ and 3′ splice sites are an important mechanism through which gene mutations cause human diseases^72,73^, including deafness^50–52^, and it has been estimated that around 15% of all disease-causing point mutations result in defective splicing. Moreover the ‘G’ nucleotide at the last base of exon present in almost 80% of the human 5′ss^74^. Interestingly, another *EYA4* variation, c.804G > C affecting the last nucleotide of exon 10, was also initially classified as a missense mutation (p.Gln268His). Subsequently, *in vitro* experiments have demonstrated that it leads to exon 10 skipping resulting in a frameshift that introduces a premature stop codon^31^. These results suggest that for some predicted *EYA4* missense mutations, the hidden mechanism of pathogenesis is rather haploinsufficiency as most mutations identified so far are frameshifts and nonsense predicted to trigger nonsense-mediated mRNA decay (NMD)^75^. Based on this hypothesis, the nonsense c.1601C > G mutation could provoke a complete degradation of the mutant messenger by NMD. Our RT-PCR experiments have shown that there were no differences in the expression levels of mutant and Wt mRNAs, however, we could not detect the p.Ser534* mutant protein by western blot. Moreover, the minigene assay has shown that this mutation does not result in an alternative splicing. Although this assay is not able to detect NMD because the generated minigene only contained one exon (exon 17); we propose that protein degradation, alone or in combination with NMD, as the possible underlying mechanism of pathogenesis of this mutation. In a previous study, Zhang and co-workers^55^ showed the same effect for the p.Arg587* *EYA4* mutation, further supporting the notion of haploinsufficiency as the pathogenic mechanism. Finally, we have identified the c.1282-1G > A mutation in family S856. This mutation destroys the acceptor splice-site of intron 14 leading to skipping of exon 15 as shown by the minigene assay and is predicted to generate a truncated protein. Hitherto, this is the second DFNA10-causing mutation affecting the acceptor splice-site of intron 14 in *EYA4*. The first mutation, c.1282-12T > A has been previously detected in an Australian family^41^.

It is of key interest the results obtained for the silent mutation c.1281G > A (p.Glu427Glu) as it is classified as “Likely pathogenic” according to the ACMG criteria by altering the donor site of intron 14, most probably affecting the splicing (Table 2); a point confirmed by the minigene assay (Fig. 8). Albeit additional experiments are further required to fully understand the mechanism of pathogenesis of this variant, to the best of our knowledge this can represent the first silent mutation to be linked to DFNA10-related hearing loss. Therefore, our findings emphasize the need to thoroughly investigate silent mutations during the filtering and prioritization of NGS variants.

Taken together, our results further support that haploinsufficiency maybe the major mechanism by which *EYA4* mutations (nonsense, frameshift, splice site and even silent mutations) cause ADNSHL. For missense mutations not affecting splicing, we also propose haploinsufficiency as the pathogenic mechanism by mean of reduced protein activity, although more experiments (e.g. to detect EYA4 level in nuclear and cytoplasmic components, respectively) are necessary to investigate whether the identified variants could affect the nuclear localization of *EYA4*. In addition, it will be interesting to generate knock-in murine models for these variants and perform RNA-seq studies in the inner ear to investigate whether the identified variants could affect transcription levels of some genes/pathways helping to fully understand the underlying mechanism of pathogenesis linked to DFNA10. In summary, we have identified nine novel DFNA10-associated variants in 8 Spanish families and one Australian family. In doing so we further illuminated the pathomechanism underlying *EYA4*-related hearing loss.

## Supplementary information

Supplementary Information.

## References

[1] Marazita ML (1993). Genetic epidemiological studies of early-onset deafness in the U.S. school-age population. Am. J. Med. Genet..

[2] Petit C (1996). Genes responsible for human hereditary deafness: symphony of a thousand. Nat. Genet..

[3] Van Camp, G. & Smith, R. J. Hereditary Hearing Loss Homepage, http://hereditaryhearingloss.org (2020).

[4] Borsani G (1999). *EYA4*, a novel vertebrate gene related to Drosophila eyes absent. Hum. Mol. Genet..

[5] Ohto H (1999). Cooperation of Six and Eya in activation of their target genes through nuclear translocation of Eya. Mol. Cell. Biol..

[6] Sadatomi D, Tanimura S, Ozaki K, Takeda K (2013). Atypical protein phosphatases: emerging players in cellular signalling. Int. J. Mol. Sci..

[7] Schönberger J (2005). Mutation in the transcriptional coactivator *EYA4* causes dilated cardiomyopathy and sensorineural hearing loss. Nat. Genet..

[8] Wang L (2008). *EYA4* regulation of Na+/K+-ATPase is required for sensory system development in zebrafish. Development..

[9] Tadjuidje E, Hegde RS (2013). The Eyes Absent proteins in development and disease. Cell Mol Life Sci..

[10] Pignoni F (1997). The eye-specification proteins So and Eya form a complex and regulate multiple steps in Drosophila eye development. Cell..

[11] Hanson IM (2001). Mammalian homologues of the Drosophila eye specification genes. Semin. Cell Dev. Biol..

[12] Jemc J, Rebay I (2007). The eyes absent family of phosphotyrosine phosphatases: properties and roles in developmental regulation of transcription. Annu. Rev. Boichem..

[13] Li X (2003). Eya protein phosphatase activity regulates Six1-Dach-Eya transcriptional effects in mammalian organogenesis. Nature..

[14] Chen A (1995). Phenotypic manifestations of branchio-oto-renal syndrome. Am. J. Med. Genet..

[15] Shinagawa J (2020). Prevalence and clinical features of hearing loss caused by *EYA4* variants. Sci. Rep..

[16] Neveling K (2013). A post-hoc comparison of the utility of sanger sequencing and exome sequencing for the diagnosis of heterogeneous diseases. Hum. Mutat..

[17] van Beelen E (2016). Audiometric characteristics of a Dutch DFNA10 Family with mid-frequency hearing impairment. Ear Hear..

[18] Frykholm C (2015). Phenotypic variability in a seven-generation Swedish family segregating autosomal dominant hearing impairment due to a novel *EYA4* frameshift mutation. Gene..

[19] Huang A, Yuan Y, Liu Y, Zhu Q, Dai P (2015). A novel *EYA4* mutation causing hearing loss in a Chinese DFNA family and genotype-phenotype review of *EYA4* in deafness. J. Transl. Med..

[20] Wayne S (2001). Mutations in the transcriptional activator *EYA4* cause late-onset deafness at the DFNA10 locus. Hum. Mol. Genet..

[21] Makishima T (2007). Nonsyndromic hearing loss DFNA10 and a novel mutation of *EYA4*: evidence for correlation of normal cardiac phenotype with truncating mutations of the Eya domain. Am. J. Med. Genet. A..

[22] Pfister M (2002). A 4-bp insertion in the eyahomologous region (eyaHR) of *EYA4* causes hearing impairment in a Hungarian family linked to DFNA10. Mol. Med..

[23] Choi HS, Kim AR, Kim SH, Choi BY (2016). Identification of a novel truncation mutation of *EYA4* in moderate degree hearing loss by targeted exome sequencing. Eur. Arch. Otorhinolaryngol..

[24] Iwasa YI, Nishio SY, Usami SI (2016). Comprehensive genetic analysis of Japanese autosomal dominant sensorineural hearing loss patients. PLoS One..

[25] Baek JI (2012). Targeted massive parallel sequencing: the effective detection of novel causative mutations associated with hearing loss in small families. Orphanet J. Rare Dis..

[26] Kim YR (2015). Evaluation of the contribution of the *EYA4* and *GRHL2* genes in Korean patients with autosomal dominant non-syndromic hearing loss. PLoS One..

[27] Abe S, Takeda H, Nishio SY, Usami SI (2018). Sensorineural hearing loss and mild cardiac phenotype caused by an *EYA4* mutation. Hum. Genome Var..

[28] Hu, S. *et al*. Genetic etiology study of ten Chinese families with nonsyndromic hearing loss. *Neural Plast*. 4920980, 10.1155/2018/4920980 (2018). 10.1155/2018/4920980 PMC6079373 30123251

[29] Sloan-Heggen CM (2016). Comprehensive genetic testing in the clinical evaluation of 1119 patients with hearing loss. Hum. Genet..

[30] Liu F (2015). Exome Sequencing Identifies a Mutation in *EYA4* as a novel cause of autosomal dominant non-syndromic hearing loss. PLoS One..

[31] Varga L (2019). Novel *EYA4* variant in Slovak family with late onset autosomal dominant hearing loss: a case report. BMC Med. Genet..

[32] Miszalski-Jamka, K. *et al*. Novel genetic triggers and genotype-phenotype correlations in patients with left ventricular noncompaction. *Circ. Cardiovasc. Genet*. **10**, 10.1161/CIRCGENETICS.117.001763 (2017). 10.1161/CIRCGENETICS.117.001763 PMC5665372 28798025

[33] Choi BY (2013). Diagnostic application of targeted resequencing for familial nonsyndromic hearing loss. PLoS One..

[34] Truong BT (2019). Exome sequencing reveals novel variants and unique allelic spectrum for hearing impairment in Filipino cochlear implantees. Clin. Genet..

[35] Sommen M (2016). DNA diagnostics of hereditary hearing loss: a targeted resequencing approach combined with a mutation classification system. Hum. Mutat..

[36] Cesca F (2018). A novel mutation of the *EYA4* gene associated with post-lingual hearing loss in a proband is co-segregating with a novel *PAX3* mutation in two congenitally deaf family members. Int. J. Pediatr. Otorhinolaryngol..

[37] Tan M (2014). Identification of I411K, a novel missense *EYA4* mutation causing autosomal dominant non-syndromic hearing loss. Int. J. Mol. Med..

[38] Sun Y (2015). A novel mutation of *EYA4* in a large Chinese family with autosomal dominant middle-frequency sensorineural hearing loss by targeted exome sequencing. J. Hum. Genet..

[39] Xiao SY (2019). Identification of a novel missense eya4 mutation causing autosomal dominant non-syndromic hearing loss in a chinese family. Cell Mol. Biol. (Noisy-le-grand)..

[40] Chen S (2016). Targeted Next-Generation Sequencing Successfully Detects Causative Genes in Chinese Patients with Hereditary Hearing Loss. Genet. Test. Mol. Biomarkers..

[41] Hildebrand MS (2007). A novel splice site mutation in EYA4 causes DFNA10 hearing loss. Am. J. Med. Genet. A..

[42] Vona B (2014). Targeted next-generation sequencing of deafness genes in hearing-impaired individuals uncovers informative mutations. Genet. Med..

[43] Cirino, A. L. *et al*. A comparison of whole genome sequencing to multigene panel testing in hypertrophic cardiomyopathy patients. *Circ. Cardiovasc. Genet*. **10** (2017). 10.1161/CIRCGENETICS.117.001768 PMC5683423 29030401

[44] De Leenheer EM (2002). DFNA10/*EYA4* the clinical picture. Adv. Otorhinolaryngol..

[45] Dutrannoy V (2009). De novo 9 Mb deletion of 6q23.2q24.1 disrupting the gene *EYA4* in a patient with sensorineural hearing loss, cardiac malformation, and mental retardation. Eur. J. Med. Genet..

[46] Schönberger J (2000). Dilated cardiomyopathy and sensorineural hearing loss: a heritable syndrome that maps to 6q23-24. Circulation..

[47] Abe Y (2009). *EYA4*, deleted in a case with middle interhemispheric variant of holoprosencephaly, interacts with SIX3 both physically and functionally. Hum. Mutat..

[48] Gana S (2019). Familial interstitial 6q23.2 deletion including *EYA4* associated with otofaciocervical syndrome. Front. Genet..

[49] Huygen PLM, Pennings RJE, Cremers CWRJ (2003). Characterizing and distinguishing progressive phenotypes in nonsyndromic autosomal dominant hearing impairment. Audiol. Med..

[50] Azaiez H (2014). *TBC1D24* mutation causes autosomal-dominant nonsyndromic hearing loss. Hum. Mutat..

[51] Azaiez H. *et al*. HOMER2, a stereociliary scaffolding protein, is essential for normal hearing in humans and mice. *PLoS Genetics*. **11**, 10.1371/journal.pgen.1005137 (2015). 10.1371/journal.pgen.1005137 PMC4376867 25816005

[52] Booth KT, Kahrizi K, Najmabadi H, Azaiez H, Smith RJ (2018). Old gene, new phenotype: splice-altering variants in CEACAM16 cause recessive non-syndromic hearing impairment. J. Med. Genet..

[53] Shearer AE (2014). Copy number variants are a common cause of non-syndromic hearing loss. Genome Med..

[54] Hildebrand MS (2011). DFNA8/12 caused by *TECTA* mutations is the most identified subtype of nonsyndromic autosomal dominant hearing loss. Hum. Mutat..

[55] Zhang Y, Knosp BM, Maconochie M, Friedman R, Smith RJH (2004). A comparative study of Eya1 and *EYA4* protein function and its implication in branchio-oto-renal syndrome and DFNA10. JARO..

[56] Morín M (2009). *In vivo* and *in vitro* effects of two novel gamma-actin (*ACTG1*) mutations that cause DFNA20/26 hearing impairment. Hum. Mol. Genet..

[57] Mencía A (2008). A novel KCNQ4 pore-region mutation (p.G296S) causes deafness by impairing cell-surface channel expression. Hum. Genet..

[58] Church DM (1994). Isolation of genes from complex sources of mammalian genomic DNA using exon amplification. Nat. Genet..

[59] Wildeman M, van Ophuizen E, den Dunnen JT, Taschner PE (2008). Improving sequence variant descriptions in mutation databases and literature using the Mutalyzer sequence variation nomenclature checker. Hum. Mutat..

[60] Richards S (2015). ACMG Laboratory Quality Assurance Committee. Standards and guidelines for the interpretation of sequence variants: a joint consensus recommendation of the American College of Medical Genetics and Genomics and the Association for Molecular Pathology. Genet. Med..

[61] Kopanos C (2019). VarSome: the human genomic variant search engine. Oxford Bioinformatics..

[62] Desmet FO (2009). Human Splicing Finder: an online bioinformatics tool to predict splicing signals. Nucleic Acid Research..

[63] Konrad, J. *et al*. Variation across 141,456 human exomes and genomes reveals the spectrum of loss-of-function intolerance across human protein-coding genes. *bioRxiv*., 10.1101/531210 (2019).

[64] Smith PJ (2006). An increased specificity score matrix for the prediction of SF2/ASF-specific exonic splicing enhancers. Hum. Mol. Genet..

[65] Cartegni L, Wang J, Zhu Z, Zhang MQ, Krainer AR (2003). ESEfinder: a web resource to identify exonic splicing enhancers. Nucleic Acid Res..

[66] Yeo G, Burge CB (2004). Maximum entropy modelling of short sequence motifs with applications to RNA splicing signals. J. Comput. Biol..

[67] Reese MG, Eeckman FH, Kulp D, Haussler D (1997). Improved splice site detection in Genie. J. Comp. Biol..

[68] Brunak S, Engelbrecht J, Knudsen S (1991). Prediction of human mRNA donor and acceptor site from DNA sequence. J. Mol. Biol..

[69] Mencía A (2009). Mutations in the seed region of human miR-96 are responsible for nonsyndromic progressive hearing loss. Nat. Genet..

[70] Baker DH, Czarnecki-Maulden GL (1987). Pharmacologic role of cysteine in ameliorating or exacerbating mineral toxicities. J. Nutr..

[71] Cartegni L, Chew SL, Krainer AR (2002). Listening to silence and understanding nonsense: exonic mutations that affect splicing. Nat. Rev. Genet..

[72] Buratti E (2007). Aberrant 5′ splice sites in human disease genes: mutation pattern, nucleotide structure and comparison of computational tools that predict their utilization. Nucleic Acids Res..

[73] Vorechovsky I (2006). Aberrant 3′ splice sites in human disease genes: mutation pattern, nucleotide structure and comparison of computational tools that predict their utilization. Nucleic Acids Res..

[74] Carmel I, Tal S, Vig. I, Ast G (2004). Comparative analysis detects dependencies among the 5_ splice-site positions. RNA..

[75] Schoenberg DR, Maquat LE (2012). Regulation of cytoplasmic mRNA decay. Nat. Rev. Genet..

