# Supplementary material for: Insights into the pathophysiology of DFNA10 hearing loss associated with novel EYA4 variants
Source: Sci Rep. 2020 Apr 10;10:6213. doi: 10.1038/s41598-020-63256-5 (PMC7148344; doi:10.1038/s41598-020-63256-5)
Supplement: Supplementary file 1 — Supplementary Information. [file 41598_2020_63256_MOESM1_ESM.pdf]

Insights into the pathophysiology of DFNA10 hearing loss associated with novel EYA4 variants

Matias Morín[1], Lucía Borreguero[1], Kevin T Booth[2,3], María Lachgar[1], Patrick Huygen[4], Manuela Villamar[1], Fernando Mayo[1], Luis Carlos Barrio[5], Luciana Santos Serrão de Castro[1], Carmelo Morales[6], Ignacio del Castillo[1], Beatriz Arellano[7], Dolores Tellería[1], Richard JH Smith[2], Hela Azaiez[2]; M.A. Moreno-Pelayo[1]*

1 Servicio de Genética, Ramón y Cajal Institute of Health Research (IRYCIS) and Biomedical Network Research Centre on Rare Diseases (CIBERER), 28034 Madrid, Spain.

2 Molecular Otolaryngology and Renal Research Laboratories, Department of Otolaryngology, Head & Surgery, University of Iowa, Iowa City, Iowa, 52242, USA.

3 Harvard Medical School, Department of Neurobiology, Boston Massachusetts, 02115.

4 Department of Otorhinolaryngology, Radboud University Nijmegen Medical Centre, Nijmegen, Netherlands.

5 Departamento de Investigación, Ramón y Cajal Institute of Health Research (IRYCIS), Unidad de Neurología Experimental, 28034 Madrid, Spain.

6 Servicio de Otorrinolaringología, Hospital Universitario Marqués de Valdecilla, 39008 Santander, Spain.

7 Servicio de Otorrinolaringología, Hospital Universitario Puerta de Hierro, Majadahonda, 28922 Madrid, Spain.

*Corresponding author:

Dr. Miguel Angel Moreno-Pelayo

Servicio de Genética-Hospital Ramón y Cajal,

Ctra. Colmenar Km 9, 28034-Madrid-Spain

Phone: 00-34-91-3368542

mmorenop@salud.madrid.org

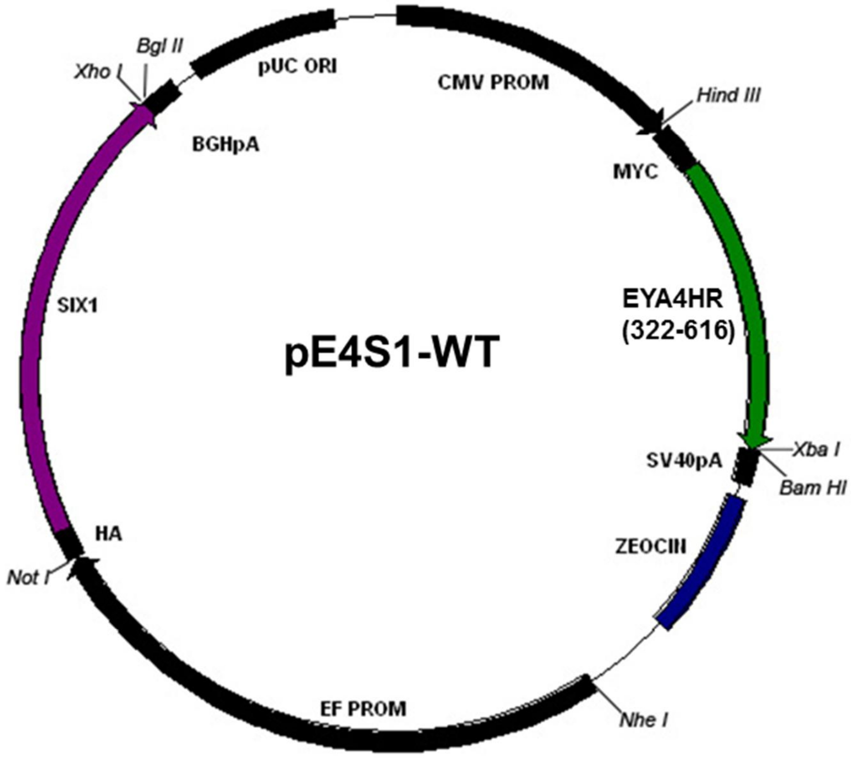

SUPL FIG1. Diagram of the bicistronic plasmid pE4S1. This vector contains the c-myc-tagged *EYA4*-HR DNA fragment that encodes from amino acids 322 until 616 of the HR domain under control of the CMV promoter and the HA-tagged Six1 protein under the EF-1 promoter.

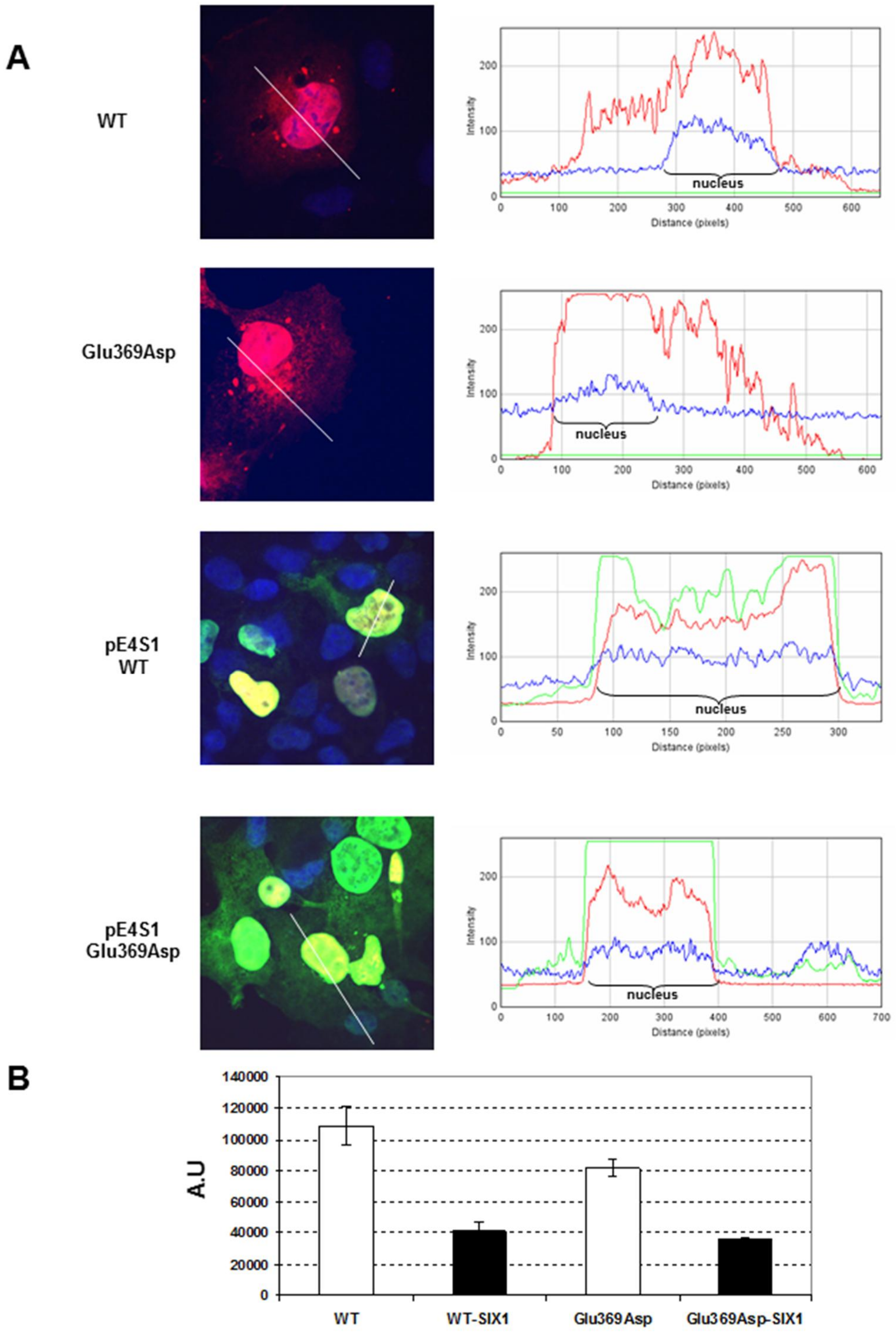

SUPL FIG2. A) Spatial distribution of *EYA4* in the absence and presence of Six 1. Image J sectional graphic analysis showing the fluorescence intensity plotted against the distance. In the presence of Six 1 both, the pE4S1-wt and the mutant pE4S1-Glu369Asp protein complexes are mainly detected in the nucleus (green and red lines). **B)** The cytoplasmic expression of *EYA4*-HR protein was significantly reduced in the presence of Six1 in both the wt and Glu369Asp proteins (black bars) when compared with the intensity obtained in the absence of Six 1 (white bars).

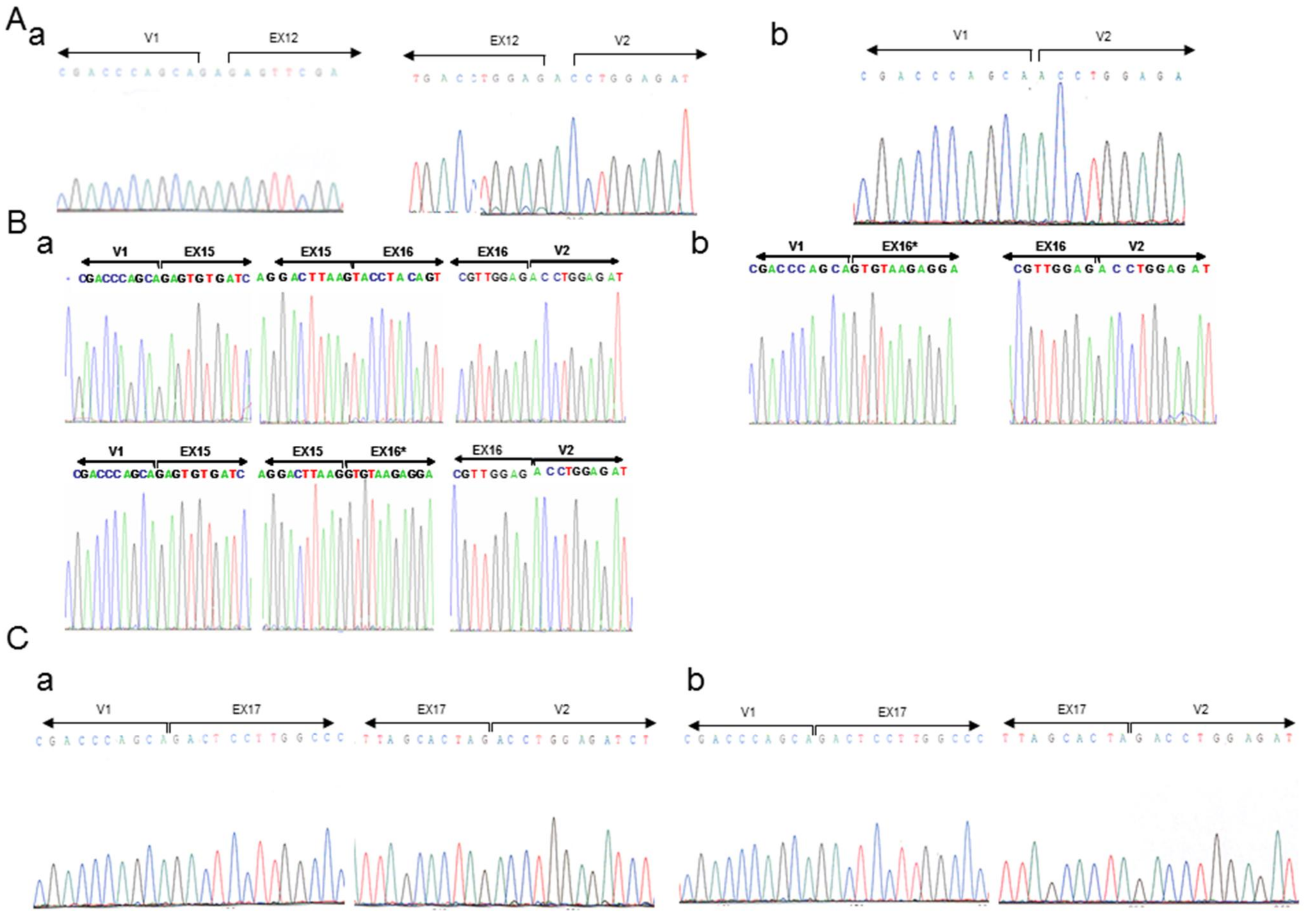

SUPL FIG3. Electropherograms of *EYA4* RT-PCR products obtained from NIH3T3 cells transfected with the minigene constructs. The exon junctions are indicated in each case. **A)** exon 12 Wt (a) and c.1107G>T (p.Glu369Asp) (b). **B)** exons 15-16 Wt (a) and c.1282 -1G>A (b). **C)** exon 17 Wt (a) and c.1601C>G (Ser534*) (b). Ex12, Ex15, Ex16, Ex17 and Ex16* of EYA4 gene denote exons 12, 15, 16, 17 and 16 lacking the first 68 pb, respectively. V1 and V2 denote the artificial exons of the pSPL3 vector.

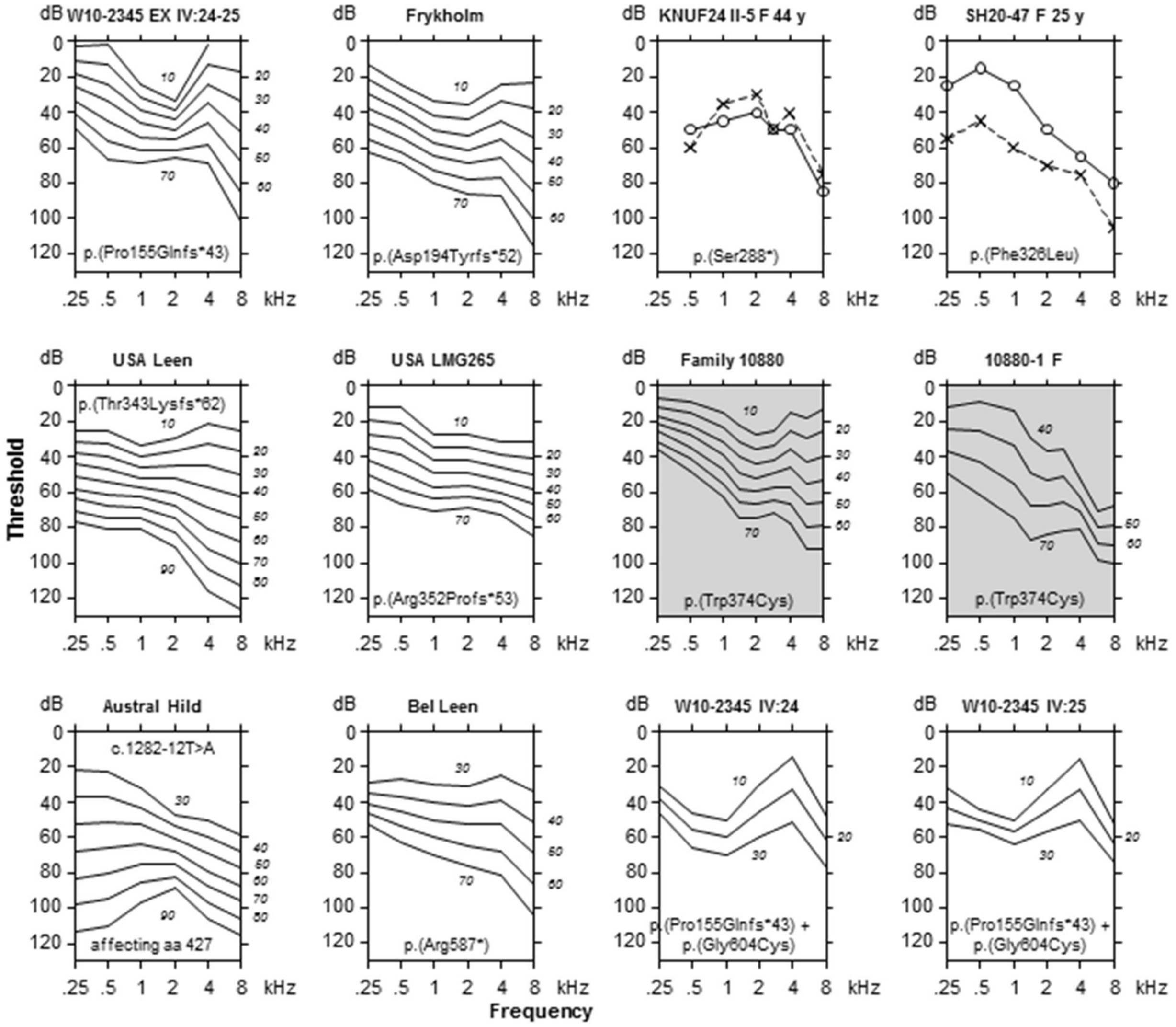

SUPL FIG4. Collage of the ARTA that were available for, or could be derived from, DFNA10/*EYA4* traits for which audiograms have been documented. Most of these ARTA demonstrate a fairly similar picture.

A

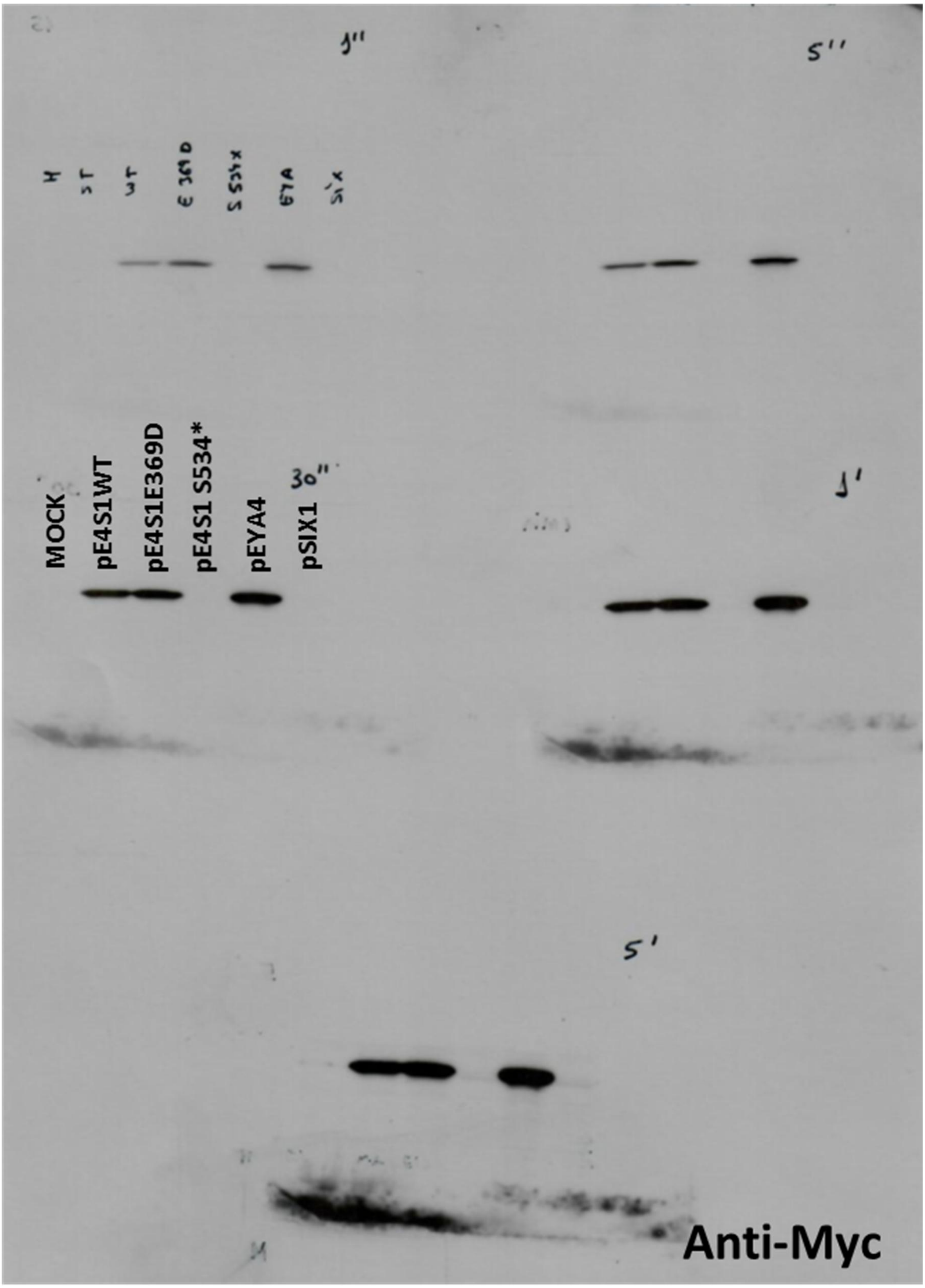
MOCK
pE4S1WT
pE4S1E369D
pE4S1 S534*
pEYA4
pSIX1
30''
5''
5'
Anti-Myc

B

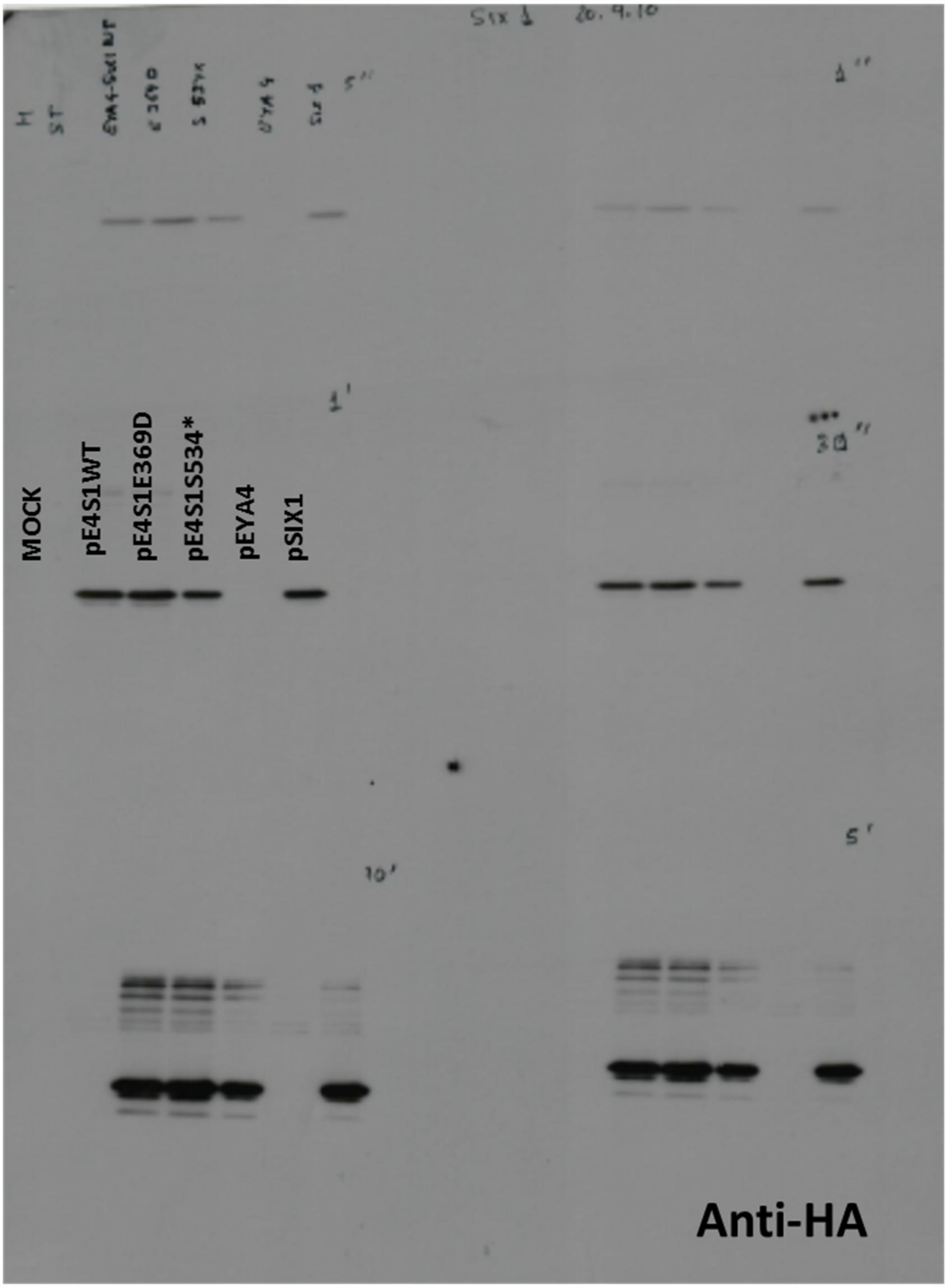

SUPL FIG5. Western blot analysis of *EYA4* Wt and mutants' production. The full-length blots at different time exposures have been displayed. **A)** A clear band of 37 kDa is observed in extracts from COS7 cells transfected with pE4S1 Wt and Glu369Asp bicistronic plasmid and in the control pEYA4 revealed with anti-Myc antibodies. No signal was detected in the mutant Ser534* when we use anti-Myc antibodies. **B)** A robust band of 33kDA corresponding to Six1 was detected when we use anti-HA antibodies (at the bottom). This band was not detected in cells transfected with the control *EYA4* plasmid.
